# Multicellularity and the Need for Communication—A Systematic Overview on (Algal) Plasmodesmata and Other Types of Symplasmic Cell Connections †

**DOI:** 10.3390/plants12183342

**Published:** 2023-09-21

**Authors:** Linus Wegner, Merlin Leon Porth, Katrin Ehlers

**Affiliations:** Institute of Botany, Justus-Liebig University, D-35392 Giessen, Germany; merlin.l.porth@bio.uni-giessen.de

**Keywords:** algae, Chlorophyta, cell connections, evolution, multicellularity, Phaeophyceae, plasmodesmata, Rhodophyta, Streptophyta, Volvocaceae

## Abstract

In the evolution of eukaryotes, the transition from unicellular to simple multicellular organisms has happened multiple times. For the development of complex multicellularity, characterized by sophisticated body plans and division of labor between specialized cells, symplasmic intercellular communication is supposed to be indispensable. We review the diversity of symplasmic connectivity among the eukaryotes and distinguish between distinct types of non-plasmodesmatal connections, plasmodesmata-like structures, and ‘canonical’ plasmodesmata on the basis of developmental, structural, and functional criteria. Focusing on the occurrence of plasmodesmata (-like) structures in extant taxa of fungi, brown algae (Phaeophyceae), green algae (Chlorophyta), and streptophyte algae, we present a detailed critical update on the available literature which is adapted to the present classification of these taxa and may serve as a tool for future work. From the data, we conclude that, actually, development of complex multicellularity correlates with symplasmic connectivity in many algal taxa, but there might be alternative routes. Furthermore, we deduce a four-step process towards the evolution of canonical plasmodesmata and demonstrate similarity of plasmodesmata in streptophyte algae and land plants with respect to the occurrence of an ER component. Finally, we discuss the urgent need for functional investigations and molecular work on cell connections in algal organisms.

## 1. Multicellularity and the Need of Communication

The evolution of eukaryotic organisms has generated multicellular body plans in several lineages. It is assumed that at least 25 independent events enabled the initial transition from unicells to simple multicellular forms such as small aggregates of genetically non-related cells, clonal coenobia/colonies, or unbranched clonal filaments [1,2,3,4,5]. Yet, recent work suggests even more of these transitional events exist [6,7].

Even simple multicellularity requires physical cell–cell adhesion [2,5,8,9,10], causing energetic efforts for the synthesis of the adhesion molecules. However, selective advantages of simple multicellularity have been validated experimentally [11,12] or via computational modelling [13,14]. They include size-dependent protection from predation [15,16], metabolic cooperation for improved acquisition of resources [17], higher stress resistance [18], and many more.

Moreover, simple multicellularity paves the way for the development of derived body plans and complex multicellularity characterized by intercellular cooperation and communication, complex 2-D and 3-D growth, cell internalization and separation from the environment, as well as cell-type differentiation [2,3,5,19]. This development includes polarized and localized growth, asymmetric cell divisions, and rotations of division planes [20]. Moreover, subpopulations of cells within the multicellular organisms undergo distinct developmental fates [21] facilitating division of labor and specialization of cell and tissue types, e.g., absorbing and protective epithelia, long-distance transport systems, storage tissues, etc.

It should be taken into account, however, that less complex body plans and even a lack of multicellularity might be advantageous under particular circumstances, as it would guarantee, e.g., maximum contact of the organism to the resources in the environment, rapid life cycles, and higher mutation rates [2]. Thus, loss of complexity and/or multicellularity might also have occurred at several points in the evolution of eukaryotes.

As stated by Niklas and Newman 2013 [4,5], the typical way to achieve multicellularity would be a transition series from unicellular—via colonial—to multicellular body plans, but an alternative way via siphonous intermediates should also be considered. Recent theories describe the evolution of multicellularity as one of the central ‘evolutionary transitions in individuality’ [22]. In a first step, unicells group together, whose cell traits are adapted to unicellular life, since they experience natural selection as lower-level entities (Darwinian particles). Cells within these collectives are exposed to an altered environment evoking new tradeoffs which might be optimized within the constraints of the cell traits. In the next step, the collectives may transform into functionally integrated, multicellular Darwinian individuals, being subjects of reproduction and selection processes as higher-level entities. The latter step includes the evolution of novel hereditary group-level traits giving rise to tradeoff breaking and multicellular adaptation [23,24].

Cooperative cellular activities of multicellular organisms (e.g., synchronous movement), temporally and spatially coordinated development (cell division, differentiation, and patterning), and collaborative physiology (metabolism, responses to stimuli and stresses) require sophisticated mechanisms controlling the cellular interactions on the molecular level [1,2]. Furthermore, it requires elaborate systems for cell-to-environment and cell-to-cell communication via ligand-receptor-mediated signaling [25,26], extracellular vesicles [27,28,29,30], and direct cytoplasmic intercellular connections, which might be regarded as a typical group-level trait.

**Figure 1:**
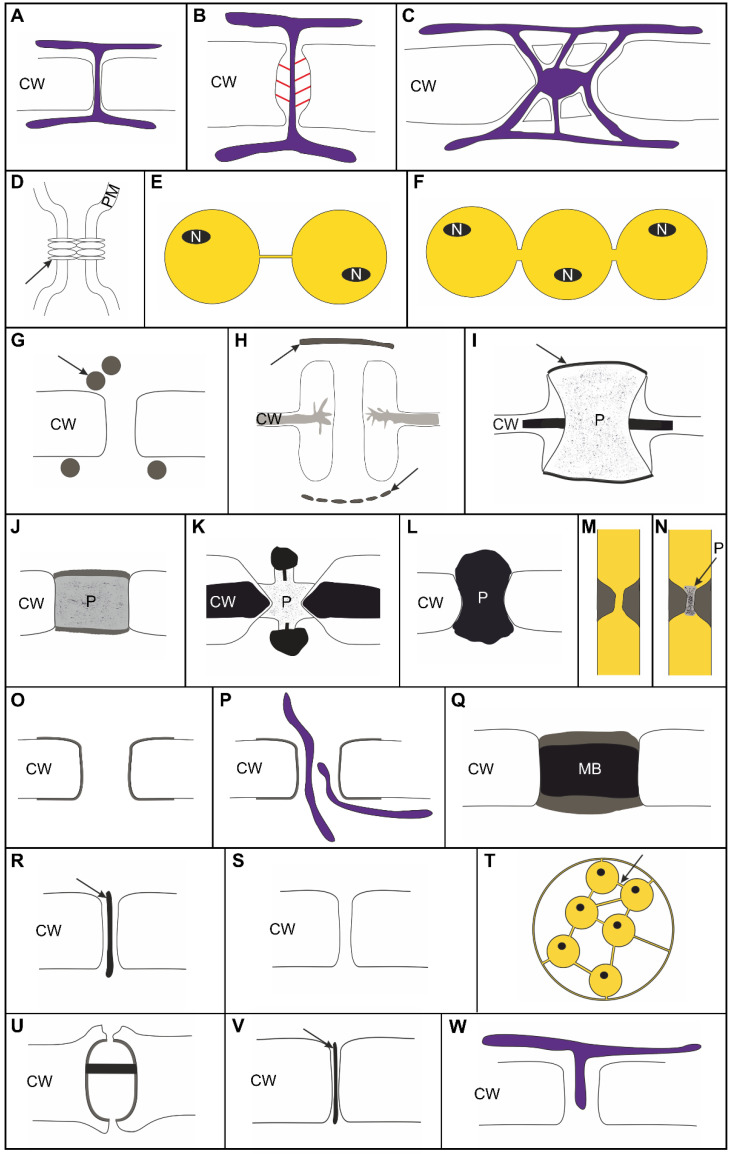
Diversity of cell connections. Canonical PD possess an ER-derived fixed desmotubule. (**A**) Young PD lack a visible cytosolic sleeve (Type I PD), while (**B**) older PD (Type II PD) show a visible cytosolic sleeve and the desmotubule is fixed by spoke-like tether proteins (red) [32,34]. (**C**) Old PD may also form complex branched structures with multiple interconnected orifices [32]. (**D**) Animals interconnect cells symplasmically via small protein-based gap junctions (arrow: connexin hexamer) [41], (**E**) membrane-lined tunnelling nano tubes (TNTs; N: nucleus) [42], and (**F**) larger intercellular bridges persisting postcytokinetically [43]. (**G**) Simple fungal septal pore associated with Woronin bodies (arrow) [44,45]. (**H**) More complex fungal dolipore (arrows: parenthesomes) [46]. Large single plugged pores of red algae (**I**) with and (**J**) without plug caps (arrow) [47]. Similar plugged pores in (**K**) *Smithsoniella earleae* (Chlorophyta) [48] and (**L**) *Ctenocladus circinnatus* (Ulvales, Ulvaphyceae) [49]. Siphonous *Codium* sp. (Bryopsidales, Ulvophyceae) forms (**M**) cytosolic pores and (**N**) plugs them [50]. Developing *Volvox* sp. (Chlamydomonadales, Chlorophyceae) colonies form (**O**) membrane-lined cytoplasmic bridges coated with a dense material of unknown nature (grey), which might be regarded as PDLCs and (**P**) are often traversed by ER strands [51]. Some species keep (**Q**) persistent cytoplasmic bridges at maturity which contain disc-shaped, layered medial bodies [52]. (**R**) PDLCs of some fungi and Chaetophoraceae species possess a central structure (arrow) that is not connected to the ER [53]. (**S**) More common algal PDLCs—e.g., in Phaeophyceae [54], Trentepohliales (Ulvophyceae) [49] and Chaetophorales (Chlorophyceae) [55,56]—are simple membrane-lined channels without an inner structure. (**T**) In the siphonocladous species *Valonia ventricosa* (Cladophorales, Ulvophyceae), the vacuole separates uninucleate domains which are interconnected by small cytoplasmic strands (arrow) [57]. (**U**) The PDLC of Oedogoniales are very different in size and shape. Globular particles are aligned at the membrane (grey) and an electron-dense band crosses the channel [58]. (**V**) Thin PDLCs of *Coleochaete scutata* (Coleochaetophyceae, Streptophyta) are traversed by a central structure without ER connection (see Section 4.5). (**W**) Temporary unilateral ER penetration in *Chara vulgaris* (Charophyceae, Streptophyta) PDLCs [59]. ER/desmotubule: violet, spoke-like tethers: red, CW: cell wall, MB: medial bodies, N: nucleus, P: plug, PM: plasma membrane.

In this review, we depict the diversity of direct cytoplasmic cell connections in multicellular organisms of the eukaryotic lineages animals, fungi, red algae, brown algae, and green algae (Chlorophyta and streptophyte algae) which have evolved independently from each other to provide pathways for the immediate cytosolic exchange of signaling molecules and metabolites. Most of these cell connections also establish direct plasma-membrane contacts to enable membrane flow and electrical coupling between cells. The structure, functioning, and mechanism(s) of formation of the diverse types of cell connections are compared to the sophisticated plasmodesmata connections (PD, Figure 1A–C) of land plants, which have intensely been studied in angiosperms and are known to play significant roles in plant development, physiological acclimation, and adaptation, as well as in pathogen response [31,32,33,34,35,36]. PD have the potential to interconnect the cytoplasm of adjacent plant cells to a synchronized, superordinated symplasmic continuum, since they convey diffusional transport of small molecules as well as targeted symplasmic exchange of non-cell-autonomous macromolecules like RNAs and proteins including transcription factors [32,37]. Moreover, PD do not only link the plasma membranes of adjacent cells, but they also connect their endomembrane systems via the desmotubule, a central ER-derived substructure (Figure 1 A–C). Remarkably, PD connectivity is highly dynamic, since PD numbers (and structures) can be adapted to changing requirements and PD permeability can be precisely controlled by distinct mechanisms [31,32,34,38]. Complex patterns of symplasmic continuity and discontinuity are established in the plant tissues and subdivide the multicellular plant organism into a dynamic system of cooperating symplasmic domains [31,39,40].

## 2. Please Contact—Various Types of Non-Plasmodesmatal Cell Connections

It appears plausible that structures with functional analogies to land-plant plasmodesmata (PD) occur in multicellular organisms of all major eukaryotic taxa (and have even been found with bacteria) [60], although these structures show obvious morphological and biochemical differences when compared to ‘canonical’ PD (Figure 1A–C) [32,34].

### 2.1. Animals

Functional analogies occur between PD and the gap junctions of animal cells (Figure 1D) [41,61,62] which mediate electrical coupling as well as symplasmic intercellular transport (or export [62]) of molecules smaller than 1.2 kDa. Gap junction permeability is regulated in response to electrical and physiological signals (e.g., pH, Ca^2+^). They consist of two opposing hemichannels spanning the plasma membranes of adjacent cells. Each hemichannel is formed by homo- or heterotypic complexes of transmembrane proteins—connexin hexamers in vertebrates and innexin octamers in invertebrates [41,63]. Different connexin isoforms have a tissue- and cell-specific distribution, but, remarkably, lack similarities of amino acid sequence to innexins [41,61,62].

Tunneling nanotubes (TNTs) are another type of (transient) cell connections establishing full symplasmic connectivity even between non-clonally related animal cells (Figure 1E) [64,65,66]. They are 50–700 nm long, but thin (<1 µm), membrane-lined anastomoses which usually are formed de novo, i.e., secondarily from cellular protrusion containing F-actin filaments and sometimes also microtubules [42]. Besides electrical signaling, TNTs mediate the exchange of cytosolic factors like metabolites, ions, and signal (macro)molecules including small RNAs. Even organelles like mitochondria or vesicles traffic though these cell connections to orchestrate development, signaling, damage repair, and immune response. Yet, TNTs are also involved in (tumor) pathogenesis and infectious diseases, since viruses, bacteria, and prions exploit the symplasmic routes for spreading [67]. TNT-like structures varying slightly in morphology and function have been described for distinct cell types of diverse animal taxa (e.g., cytonemes, tumor microtubes) [65,66,68].

Much wider intercellular bridges with diameters of up to 10 µm occur transiently in various animal taxa, namely in the germline where they interconnect germ cells to ‘germ-cysts’, between somatic cells during invertebrate development, as well as between pluripotent embryonic or adult stem cells (Figure 1F) [43,65]. They develop from an incomplete cytokinesis by ingressive furrowing, leaving a single, central, residual, plasma membrane-lined symplasmic bridge open for intercellular communication between the sister cells [65]. The symplasmic bridges in other eukaryotic taxa with complex multicellular body plans discussed in the following three paragraphs exhibit the same features [43].

### 2.2. Fungi

Septal pores, found in the center of the transverse cell walls of filamentous fungi, also develop from an incomplete furrowing where a contractile actin/myosin ring drives furrow ingression (Figure 1G,H) [69], similar to animal cells [70]. Exocytotic vesicles deliver and deposit cell-wall material to the ingrowing septum [69]. Septal pores occur in the septate hyphae filaments of Basidiomycota, Ascomycota, and some taxa of the Zygomycota, but pores of different taxa vary in size (25–500 nm) and with respect to other ultrastructural features. In the Ascomycota, septal pores are simple plasma membrane-lined channels (Figure 1G), while most taxa of the Basidiomycota (e.g., Agaricomycotina) form characteristic dolipores (Figure 1H) [69,71]. These are surrounded by barrel-shaped swellings of the septum wall and are associated on either side with septal pore caps (parenthesomes) derived from the ER. Structural details of the septal pore caps serve as valid taxonomic markers [46,72,73].

Despite their structural variability, all septal pores mediate a rapid symplasmic exchange of metabolites and macromolecules driven by fast cytoplasmic streaming. Moreover, fungal organelles—including their eukaryotic nuclei—also traffic though the perforate septa [69]. Consequently, body plans of the septate hyphae might be regarded as coenocytic rather than multicellular and the term ‘pseudocells’ has sometimes been used for the hyphal (sub)compartments [74]. However, controlled sealing of septal pores might be a means to establish heterogenous regions within the fungal mycelia in order to enable polarized apical tip growth, heterogenous gene expression, and metabolic specialization [69]. A transient or permanent plugging of the septal pores also occurs in response to wounding and other abiotic stresses. Pores are either occluded with proteinaceous materials and/or organelles, or, in filamentous Ascomycota (Pezizomycotina), with the help of special peroxisome-like Woronin bodies in an ATP-dependent manner. Permanent occlusion of the septal pores is achieved by deposition of cell-wall material in a following consolidation phase [45,69,75]. Recent molecular analyses on Ascomycota identified proteins located in the septal pores and genes involved in regulating the pore plugging [76].

### 2.3. Red Algae

**Figure 2:**
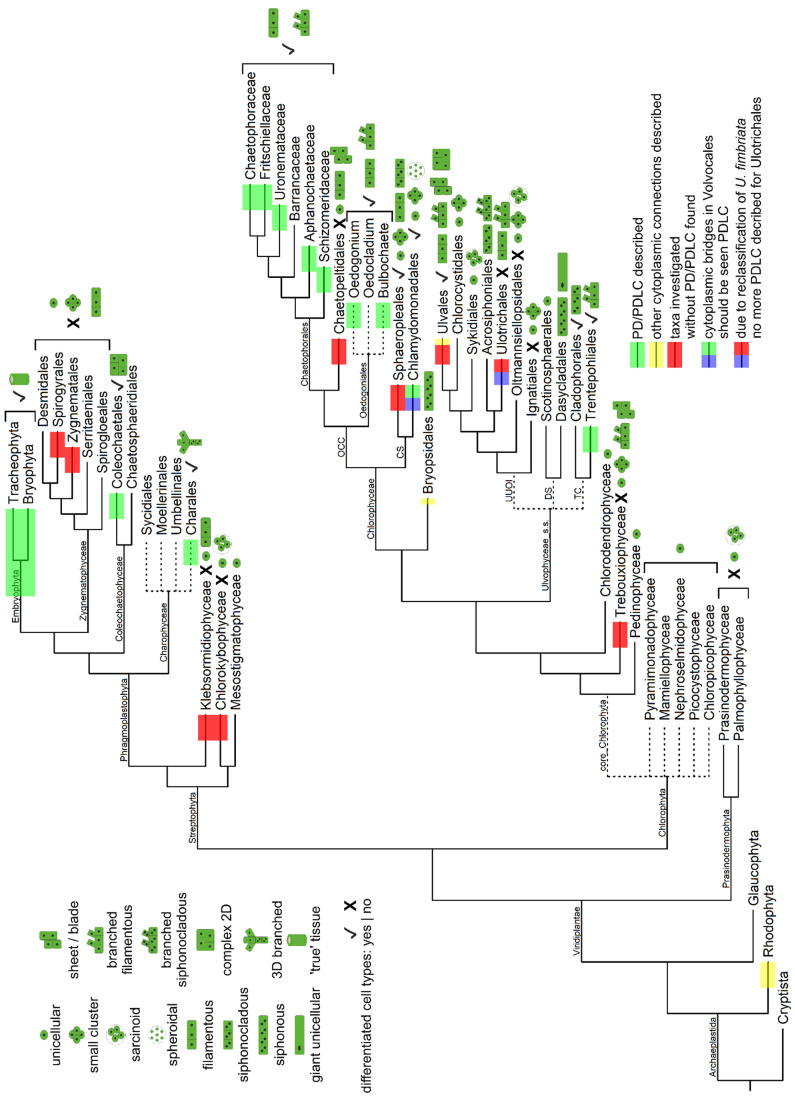
Phylogeny of the Archaeplastida (especially Viridiplantae) with focus on the occurrence of PD/PDLCs (color blocks), body plans (pictographs), and absence/presence of cell differentiation (X|✓). The phylogeny is based on [7,77,105,106,107,110,111,112,113]. Body plan information and cell differentiation status is adopted from [1,3] with additional information from [77,110,112,114,115]. Information on PD presence is based on Table 1, Table 2 and Table S1. Dotted branches indicate polytonies which are not sufficiently supported yet, or groups whose exact relationship is not essential for the topic of this review. The branch lengths do not represent phylogenetic distances.

Membrane-lined symplasmic cell connections derived from incomplete furrowing were also found within the Archaeplastida [77]. The pit connections/pit plugs of complex multicellular Rhodophyta develop in a two-phased process: (i) the maintenance of a central open cytoplasmic pore of about 1 µm during annular ingrowth of the septal wall and (ii) the subsequent formation of a conspicuous, mainly glycoproteinaceous plug which seals the pore and whose structure varies between taxa (Figure 1I,J and Figure 2) [47,78,79]. Many Florideophyceae form complex, layered pit plugs including cap membranes which are continuous with the plasma membrane of the adjacent cells and separate the plugs from the cytoplasm (Figure 1I). The assumption that the plugging material and/or the cap membranes interrupts symplasmic exchange [78,80] is called into question by the recent finding that microinjected FITC-labeled dextrans of up to 10–20 kDa traffic cell-to-cell in young filament parts of *Griffithsia monilis*, a member of the Ceramiales [81]. Unfortunately, it has not been proven in structural analyses whether the pit connections were fully mature at this developmental stage. Moreover, pit connections mediate membrane continuity between cells and electrical coupling was proven for *Griffithsia pacifica* [82].

Remarkably, there are also reports on the occurrence of secondary pit connections in Rhodophyta, namely at the interspecific cell interface of a parasitic red algae and another host Rhodophyta species [83,84,85]. It was argued that pit connections between species could certainly not develop (primarily) during cytokinesis, but must have been formed by an entirely different mechanism [31]. Similar arguments hold true for interfaces between clonally non-related cells of the same organism, e.g., the lateral walls in the pseudoparenchymatous Rhodophyta thalli [86,87]. It turned out, however, that all the putative ‘secondary’ pit connections develop during a strictly unequal, intercalary cell division of the (parasitic) ‘donor’ cell [85,87]. This division gives rise to a tiny uninucleate conjunctor cell that approaches the adjacent (host) ‘acceptor’ cell, but remains symplasmically connected to the ‘donor’ by the ‘secondary’ pit connection. During the following fusion of the conjunctor and the ‘acceptor’ cell, the conjunctor cell’s content is released into the ‘acceptor’ cell, which becomes heterokaryotic and directly linked to the ‘donor’ via the persisting ‘secondary’ pit connection.

The term ‘secondary’ pit connection is misleading, though, since for plant PD, the terms ‘primary’ and ‘secondary’ strictly pertain to the PD origin. While primary PD form during cell division when a cell plate is laid down between the clonally related daughter cells, secondary PD are formed postcytokinetically in an already existing wall between related or non-related cells [31]. According to this definition, the ‘secondary’ pit connections of the Rhodophyta are clearly primary in origin, i.e., they are formed in the course of a cell division, although they interconnect clonally non-related cells after the fusion process. To avoid terminological confusion, we suggest a neutral descriptive nomenclature to describe this type of cell connections, e.g., conjunctor pit connections.

### 2.4. Green Algae—Ulvophyceae and Chlorophyta with Uncertain Taxonomic Position

It is worth noting that, among the Archaeplastida, single, centrally located ‘septal plug’ cell connections with striking morphological similarities to the pit plugs of Rhodophyta have also been reported for the unbranched filaments of *Smithsoniella earleae*, which divides by furrowing, lacks PD, and is currently ranked as Chlorophyta *classis incertae sedis* (Figure 1K, Table S1) [48,88]. The same applies to the (plugged) pit-like connections of *Ctenocladus circinnatus*, classified as a member of the Ulvales (Ulvophyceae) which also divides by furrowing (Figure 1L and Figure 2, Table S1) [49,89]. Moreover, in a light microscopical study dating from 1933, centripetal ingrowth of transverse walls (‘Zellwandgürtel’) has regularly been observed with three species of *Codium* (Bryopsidales, Ulvophyceae) whose organisms consist of a single, multinucleate giant cell [50]. Depending on the season, these cell walls leave a central cytosolic pore with a diameter of 1.5–2 µm open between the neighboring cytoplasmic compartments, or the pores are reversibly plugged (Figure 1M,N and Figure 2, Table S1)—reminiscent of fungal septal pores and pit plugs of Rhodophyta. Permanent plugging of the pores was also observed with *Codium* and might be involved in the fragmentation of the giant cell [90].

### 2.5. Green Algae—Volvocaceae

Finally, within the Archaeplastida, cytoplasmic bridges are also formed by the Volvocaceae positioned within the Chlorophyta (Chlorophyceae, Chlamydomonadales). These are known as model organisms for the evolution of multicellularity [10,91] because they form spherical colonies which vary in cell number and complexity [92,93,94]. High numbers of cytoplasmic bridges with diameters of about 100–200 nm develop during asexual reproduction of spherical Volvocaceae colonies, when an ‘embryo’ is formed in a specialized gonidium cell of the parental colony. This occurs mostly by rapidly successive synchronous divisions (multiple fission), giving rise to a clonally related daughter colony with a defined cell number [10,51,93,95,96]. Notably, at least in the well-studied *Volvox carteri*, cell divisions follow a ‘hybrid mode’ with: (i) unilateral ingressive furrowing at the flagellar cell side and (ii) alignment of (Golgi)vesicles among phycoplast-like ‘cleavage microtubules’ at the opposite side. The latter forms a cell plate-like structure where regularly spaced bands of cytoplasmic bridges are left open between the fusing vesicles, which is reminiscent of true PD formation (Figure 1O and Figure 2 [31]). The inner face of the plasma membrane lining the cytoplasmic bridges is coated with an electron-dense material and sometimes ER cisternae and/or microtubules run though these bridges (Figure 1P) [51].

The bridge bands of all ‘embryo’ cells are aligned and presumably play a role in synchronizing cell-division activity, but—even more important—they guarantee the firm mechanic cohesion and correct alignment of the ‘embryo’ cells during the subsequent inversion process. When compared to the cellular arrangement in the adult colony, the ‘embryo’ is oriented inside-out and needs to be fully inverted to bring the inner flagellar cell ends to the outside. The driving force of this process is mediated by InvA, a kinesin-like motor protein [97], located at the bridge band regions which moves along cortical microtubules to alter the cell shape and reverse the bending of the ‘embryo’ cellular sheet [10,93,96]. Similar cytoplasmic bridges were observed in *Gonium pectorale* which only requires a partial inversion to form flattened colonies [98]. Remarkably, transformation of a single *Gonium pectorale* retinoblastoma cell-cycle regulator gene (*RB*) into the unicellular relative *Chlamydomonas reinhardtii* (*rb*-defective mutant) causes colonial growth and formation of cytoplasmic bridges indicating that simple molecular changes might be sufficient to enable the transition to multicellularity [91,99].

Once the inversion process is completed, the cells in the daughter colony build a stabilizing extracellular matrix and many Volvocaceae species simultaneously lose their cytoplasmic bridges. Maintenance of cell connections in the adult colonies only occurs in three subgroups of the *Volvox* genus, but adult cells are always interconnected by lower numbers of wider cytoplasmic bridges, suggesting that they are derived from fusion of existing cell connections [10,51,96]. Disc-shaped, layered ‘medial bodies’, sometimes traversed by ER cisternae, were frequently observed in the adult cytoplasmic bridges (Figure 1Q) [52,96] and seem to plug the symplasmic transport route just like the pit plugs of Rhodophyta do. Strikingly, however, maintenance of cytoplasmic bridges in particular species correlates with the evolution of a new mechanism of ‘embryo’ formation characterized by cell growth between the successive division cycles. Connected adult cells of the parental colony supposedly use the cytoplasmic bridges to deliver nutrients to the growing daughter colonies [96], just like the nurse cells in animal germ cysts which are symplasmically linked to the oocytes [43].

## 3. Conclusions I

(1) Intercellular bridges of animals, fungal septal pores, and pit plugs of red algae show striking similarities pertaining to their general structure and to their mechanisms of formation although they have evolved independently from each other [43]. Suppression of abscission, leaving an open, membrane-lined cytoplasmic pore in the center of an ingressive cleavage furrow, might be a simple mode to form symplasmic cell connections.
  The core molecular machinery of abscission appears to be highly conserved across eukaryotes [43] and slight molecular changes may be sufficient to convert full into incomplete abscission as shown for *Chlamydomonas reinhardtii* [99]. Repeated independent evolutionary origins of (simple) multicellularity are, therefore, highly probable.
  Cell connections retain symplasmic communication between daughter cells, but also serve cell cohesion [96] which might be relevant in early phases of the transition from uni- to multicellular body plans. Such a mechanical function may also pertain to the cell connections interconnecting non-clonally related cells across lateral walls in pseudoparenchymatous thalli of Rhodophyta.
(2) Plugs observed with fungal, red algal, Ulvophyceae, and permanent volvocaceaen cell connections also occur in PD at particular interfaces of streptophyte algae [59] and between undifferentiated cells of plant calluses [39]. Targeted occlusion has likely evolved multiple times to exert systematic size restriction on symplasmic exchange [81] and eventually creates partially or fully isolated symplasmic domains [31,59,69] which subdivide the multicellular organisms into heterogenous regions with different developmental fates and/or metabolic functions to allow division of labor.
(3) We suggest the term ‘conjunctor’ pit connections to replace the term ‘secondary’ pit connections in Rhodophyta, since they develop primarily in the course of a cell division [31] and connect clonally non-related cells only after cell fusion events.
(4) Cytoplasmic bridges formed temporarily or permanently in developing Volvocaceae colonies are slightly wider than canonical PD and do not always possess an ER-derived substructure. At least in *V. carteri*, they develop among fusing vesicles in a cellplate-like structure. In this respect, they resemble canonical land-plant PD [31] and might be regarded as PD-like connections (PDLCs) in contrast to all other types of cell connection discussed in this section (Figure 2).

## 4. Get in Touch—PD and Plasmodesmata-Like Cell Connections (PDLCs)

In this section, we will focus on those symplasmic cell connections which were termed ‘PD’ in the literature, i.e., PD and PDLCs in fungi, Phaeophyceae (Figure S2), and in the distantly related Viridiplantae with the two major groups Chlorophyta and Streptophyta (Figure 2, Table 1, Table 2 and Table S1). The latter include the embryophytes which possess canonical land-plant PD (Figure 1A–C) [32,34]. We will discuss similarities and differences with respect to the structure, origin, and functioning of PD and PDLCs in the distinct groups.

Comprehensive reviews on the occurrence of PD/PDLC structures within the eukaryotes, which also consider non-PD cell connections in fungi and red algae, gathered detailed information on the present-day distribution of PD/PDLCs in order to trace back their evolutionary history and draw conclusions on the origin and evolution of land-plant PD [80,100,101,102]. It was consistently suggested that there have been 4–6 independent origins of PD/PDLCs [103]. Since then, ongoing progress of phylogenomics has unraveled evolutionary relationships between eukaryotes far more reliably than the ‘classical’ taxonomy which is predominantly based on morphological and biochemical criteria and is obscured by convergent evolution and analogies [7,77,104,105,106,107,108,109]. This caused extensive reclassifications of many taxa (Figure S1, Table S1).

Here, we present a critical update on the occurrence of PD/PDLCs in extant members of distinct algal lineages considering recent taxonomic reclassifications and novel publications (Figure 2, Figures S1 and S2, Table 1, Table 2 and Table S1). For the algal taxa of the Viridiplantae (Table 1, Table 2 and Table S1), we give particularly detailed information on species which have been investigated for the occurrence of PD/PDLC structures, including citations of the original literature, old and present taxonomic classification (species, genus, family, order, class, phylum), strain numbers in distinct algal culture collections (if traceable), and comments (Table S1). This piece of work may serve as a tool for future work aiming to fill the information ‘gaps’, achieve a systematic survey, and answer yet open questions on the structural, functional, and molecular evolution of PD and PDLCs.

### 4.1. PD/PDLCs in Fungi

Before the distinct PD-bearing algal taxa are discussed, early studies should be mentioned which described PD/PDLC structures in particular fungal species with multiperforate septa within the Ascomycota (e.g., *Geotrichum candidum* and *Endomyces* (=*Saccharomyces*) *fibuliger*), Mucoromycota (e.g., *Rhizopus sexualis* and *Gilbertella* sp.), and Chytridiomycota (e.g., *Entophlyctis* sp. and *Rhizophydium* sp.) [100,102]. The diameter of these membrane-lined cytosolic channels (20–70 nm) resembles that of canonical PD and in some species a solid ‘core’ (Figure 1R) or even an ER-associated ‘desmotubule’ traverses the PD/PDLCs (Figure 1A). For the cytokinesis of *Rhizopus sexualis*, it was shown that the centripetally growing septum is elongated by fusion of linearly aligned vesicles just like in a centrifugally growing plant cell plate [116]. Thus, the formation of true PD among these vesicles appears feasible. However, recent studies on fungal PD/PDLC structures are scarce (for a basidiomycete [117]) and do not focus on their development or (ultra)structure.

With regard to the distant phylogenetic position of fungi and land plants, their PD/PDLCs most likely evolved from independent origins. However, it has been supposed [118] that genes required to form land-plant PD with ER components were laterally transferred to the PDLC-forming fungi, possibly via plant viruses which use fungi as transmitting vectors [119,120,121,122]. It would be interesting to analyze whether the respective fungi indeed contain orthologs of plant PD genes, e.g., ER-shaping reticulons [123] or ERplasma membrane tethers [124,125,126].

### 4.2. PD/PDLCs in Brown Algae—Phaeophyceae

The Phaeophyceae are a comparably young taxon within the stramenopiles which diverged from the direct sister group Schizocladiophyceae approx. 260 million years ago (MYA) [127] and have evolved independently from the Archaeplastida (Figure S1). Typically, they occur in marine habitats and all members are multicellular. Yet, they vary with respect to the complexity of their body plans from simple unbranched filaments to complex parenchymatous forms, some of which have achieved high levels of cell, tissue, and organ differentiation (Figure S2). Well-known examples are giant macroalgae like *Macrocystis* (Laminariales) in the marine kelp forests, which possess sieve tube-analogous, symplasmically interconnected cell arrays serving the long-distance transport of carbohydrates [128,129]. Alternating generations of the life cycle may be isomorphic in the Phaeophyceae, but often the gametophyte has a simpler or reduced body plan when compared to the respective sporophyte (Figure S2) [130].

A detailed review on the occurrence of PDLCs in the Phaeophyceae was published in 2015 [54] after the major reclassification of the taxon was proposed [131]. PDLCs of Phaeophyceae are always straight, unbranched, membrane-lined cytosolic channels with an inner diameter of 10–20 nm. Consistently, it was shown that they lack a central ER-derived desmotubule structure (Figure 1S), except for very few old reports for some Laminariales PD [100,102]. Thus, PDLCs in Phaeophyceae differ from canonical land-plant PD, but they were found in all species analyzed, so a single evolutionary origin of all Phaeophyceae PDLCs appears likely. PDLCs were found in filamentous Phaeophyceae algae as well as in pseudoparenchymatous and parenchymatous forms (Figure S2) [54]. Yet, in the morphologically complex Phaeophyceae (Scytosiphonaceae/Ectocarpales, Laminariales, Desmarestiales, Fucales and Dictyotales), PDLCs are arranged in central pit-field clusters, while they are distributed uniformly in the cell walls of taxa with simpler body plans (Ectocarpaceae/Ectocarpales and Sphacelariales) (Figure S2) [54]. Evidence for the presence of PDLCs in Ishigeales (Figure S2) comes from *Petroderma maculiforme* [132] and from herbarium specimens of the pseudoparenchymatous *Pilinia rimosa* [133], which was recently reclassified as member of the Ishigeales [134]. However, the branched filamentous thalli of *Schizocladia ischiensis*—the only member of the Schizocladiophyceae which are sister to the Phaeophyceae—lack PDLCs [135].

In all Phaeophyceae species which have recently been investigated, primary PDLCs develop cytokinetically within a patchy, linear alignment of Golgi vesicles and flat cisternae of unknown nature reminiscent of a cell plate, although furrowing was also reported in the old literature [54,136,137]. Larger ‘membrane sacs’ are built by lateral fusion of the vesicles and cisternae, and ‘preplasmodesmata’ presumably develop from plasma-membrane invaginations and cytoplasmic protrusions in these regions. Localization of the ‘preplasmodesmata’ determines the position of the mature PDLCs which develop while the membrane sacs expand laterally to form a complete ‘cell partitioning membrane’ and a young cell wall. Flat cisternae were still found in mature cells in the vicinity of all cell walls and they were densely aggregated at the pit fields, where many PDLCs are clustered [138]. It was speculated that the flat cisternae might have a general function in cell-wall deposition and maintenance and/or in PDLC transport.

Remarkably, primary formation of PDLCs does not occur in the first (and second) cell wall built by germinating zygotes of *Fucus* (Fucales) [54,139]. However, in later developmental stages of these walls, simple straight PDLCs were observed, which must have developed de novo and be of secondary origin, but cannot be distinguished from primary PDLCs based on their (sub)structure. Ongoing secondary PDLC formation during germling development leads to increasing PDLC densities, but the mechanism of secondary PDLC formation has not been unraveled yet.

Initial secondary PDLCs might develop in a preexisting wall from membrane invaginations and cytoplasmic protrusions as shown for the cytokinetic ‘preplasmodesmata’ of Phaeophyceae, and as discussed for secondary PD formation in angiosperm plants [31,140,141]. However, the occasional observation of ‘‘branched plasmodesmata that appeared to arise from the lateral preexisting ones’’ [139] suggests another mechanism in which existing PDLCs of Phaeophyceae may function as templates for the de novo formation of secondary PDLCs, reminiscent of the twinning mechanism observed with angiosperm PD [140,142,143]. H-shaped PD are intermediate stages in this process which finally gives rise to pairs of simple PD. Small groups of laterally adjacent PDLCs, which are otherwise evenly distributed in the cell walls of the Phaeophycaeae, as well as larger groups of PDLCs located in pit-field clusters [54] might also develop from such a PDLC twinning [142,143]. The interface of coalescing holdfast structures in Laminariales and Fucales is another system in which de novo formation of simple intra- and interspecific secondary PDLCs was observed in thinned wall areas [144], but details on the mechanism were not reported.

Functional tests on the PDLCs in *Halopteris congesta*, a multiseriate filamentous member of the Sphacelariales, revealed a size-exclusion limit of at least 20 kDa at the interface of apical and subapical cells [145]. In zygote-derived germlings of *Fucus distichus*, microinjected FITC-labelled dextranes of 3 kDa spread between all cells, while 10 kDa dextranes cannot pass the interface between rhizoid and thallus cells, and 40 kDa dextranes do not traffic at all. This indicates that transport through Phaeophyceae PDLCs can be controlled and adapted to distinct requirements [139]. Regulation is possibly achieved by special cell-wall pores surrounding the PDLC or by internal filament-like substructures that are sometimes found in the cytosolic channel of the Phaeophyceae PDLCs [137,139].

Although PDLCs were observed in a variety of species from seven different orders of the Phaeophyceae (Figure S2), it would be interesting to extend the investigations to the remaining orders. Those taxa which are positioned at the base of the Phaeophyceae radiation (e.g., Discosporangiales) would be of particular interest to unravel whether the occurrence of PDLC is indeed a typical and unique character of the Phaeophyceae within the Ochrophyta. It would also be interesting to learn more about other taxa which are closely or more distantly related to the Phaeophyceae and include members with multicellular body plans, like Xanthophyceae, Phaeothamniophyceae, and Chrysophyceae [5,77]. In previous reviews, it was regularly mentioned that these algal taxa lack PD [80,101,102], but original literature demonstrating a targeted search for PD/PDLCs was not mentioned.

### 4.3. PD/PDLCs in the Green Lineage—Viridiplantae (Prasinodermophyta and Unicellular Core Chlorophyta)

Early fossil records classified as green algae date from approximately 1200 MYA [114,146]. Molecular clock analyses also confirm that the Viridiplantae are an ancient taxon and extant lineages have highly diverged from each other over millions of years of evolution [109,147]. Occurrence of PD has not been investigated for a number of Chlorophyta algal classes which are positioned at the base of radiation, after a deep phylogenetic split in the green lineage had separated the phyla Streptophyta (including land plants) and Chlorophyta about 725–1200 MYA (Figure 2 and Figure S1) [109,147,148]. Presumably, PD lack in the ‘prasinophytes’ (i.e., Pyramimonadophyceae including the Pseudoscourfieldiales/Pycnococcaeae, Mamiellophyceae, and Nephroselmidophyceae), which previously also comprised the Picocystophyceae and Chloropicophyceae [149], as well as the Palmophyllophyceae including the Prasinococcales [3,77,114,150]. The absence of PD is plausible, since these classes usually comprise small naked or scaled solitary flagellates and coccoid unicells with predominantly marine or freshwater habitats. This also applies to the Pedinophyceae and the Chlorodendrophyceae, classified as core chlorophytes in modern phylogenies (Figure 2 and Figure S1) [107,110,113]. Only members of the Palmophyllophyceae [150] and the related, recently described Prasinodermophyceae [107] have reached a particular state of multicellularity where coccoid unicells may assemble loosely in a common mucilaginous matrix (sarcinoid) and even form macroscopic palmelloid thalli. However, cell connections have not been described in these two classes which were recently grouped together in the new phylum Prasinodermophyta. This taxon represents a new sister group to all other Viridiplantae and had diverged before the split of Streptophyta and Chlorophyta [107].

### 4.4. PD/PDLCs in the Green Lineage—Viridiplantae (Multicellular Chlorophyta)

#### 4.4.1. Trebouxiophyceae

Within the core Chlorophyta, multicellular organisms have most likely developed independently from each other [1] in each of the species-rich major classes Ulvophyceae, Trebouxiophyceae, and Chlorophyceae (UTC algae, Figure 2 and Figure S1), and it was even suggested that there are multiple origins of multicellularity in each of these taxa [1]. The classes differ significantly with respect to their morphology and ecology. The Trebouxiophyceae comprise algae with freshwater or terrestrial habitats, and are lichen-forming photobionts [115,151] with unicellular, sarcinoid, branched filamentous, or sheet-like body plans (Figure 2). Even the multicellular species of the Trebouxiopyceae do not show cell-type diversification and the occurrence of PD was not reported, although several investigations were performed on six genera from five distinct families classified in three different orders (Table 1 and Table S1). Thus, lack of PD in the Trebouxiophyceae is comparably well supported.

**Table 1:** Chlorophyta species which have been studied for PD/PDLC presence sorted by order, family, and genus. Numbers of examined species per genus, numbers of PD/PDLC-containing species, and numbers of relevant publications are shown. Based on Table S1.

Class	Order	Family	Genus	n (Species Examined)		n (Publications)
				Total	with PD(LC)	
Trebouxiophyceae	Prasiolales	Stichococcaceae	*Deuterostichococcus*	1	0	1
			*Stichococcus*	1	0	3
			+6			
		Koliellaceae	*Koliella*	1	0	1
			+4			
		Prasiolaceae	*Prasiola*	1	0	1
			+8			
		+1				
	Microthamniales	Microthamniaceae	*Microthamnion*	1	0	1
			+2			
	ordo i.s.	familia i.s.	*Leptosira*	1	0	2
	+4					
Ulvophyceae	Ulotrichales	Ulotrichaceae	*Ulothrix*	11	1 ^a^	6 + 2
			+20			
		Sarcinofilaceae	*Sarcinofilum*	1	0	2
			+1			
		Helicodictyaceae	*Rhexinema*	1	0	1
			+2			
		Tupiellaceae	*Vischerioclonium*	1	0	2
			+1			
		+11				
	Ulvales	Ctenocladaceae	*Ctenocladus*	1	0	3 + 1
			+3			
		Ulvaceae	*Ulva*	4	0	6
			*Percursaria*	1	0	1
			+11			
		Kornmanniaceae	*Lithotrichon*	1	0	1
			+7			
		Ulvellaceae	*Ulvella*	1	0	1
			+6			
		+3				
	Trentepohliales	Trentepohliaceae	*Trentepohlia*	7 + 4	7 + 4	9
			*Cephaleuros*	2 + 2	2 + 2	3
			*Phycopeltis*	1	1	1
			+6			
	+10					
Chlorophyceae	Chaetophorales	Aphanochaetaceae	*Aphanochaete*	2	2	2
			+3			
		Barrancaceae	*Barranca*	1	0	1
		Chaetophoraceae	*Stigeoclonium*	6 + 1	6 + 1	6
			*Caespitella*	1	1	1
			*Chaetophora*	1	1	1
			*Draparnaldia*	1 + 1	1 + 1	1 + 1
			*Gongrosira* ^b^	1	0	2
			*Pleurococcus* ^c^	1	0	1
			*Sporocladopsis*	1	1	1
			*Leptosiropsis*	1	0	1
			+42			
		Fritschiellaceae	*Fritschiella*	2	1 ^d^	2
		Schizomeridaceae	*Schizomeris*	1	1	1 + 1
		Uronemataceae	*Uronema*	3 + 1 ^e^	3 + 1 ^e^	2
	Chaetopeltidales	Chaetopeltidaceae	*Pseudulvella*	1	0	1
			*Koshicola*	1	0	1
			*Oncosaccus*	1	0	1
			+7			
		+1				
	Oedogoniales	Oedogoniaceae	*Oedogonium*	3 + 2	1 + 2	5
			*Bulbochaete*	1	1	5
			+2			
	Sphaeropleales	Microsporaceae	*Microspora*	1	0	2
			*Flintia*	1	0	1
		Cylindrocapsaceae	*Cylindrocapsa*	2	0	3
			+2			
		+20				
	+1					

i.s.: incertae sedis. ^a^ only *Ulothrix fimbriata* with PDLCs which should be reclassified as *Uronema fimbriata* (Figure S3). ^b^ another *Gongrosira* (*G. fluminensis*) species was recently reclassified as Ulvales [152]. ^c^ *Pleurococcus* sp. cannot unambiguously be identified; many species were reclassified since 1973. ^d^ McBride 1970 [153] is commonly cited for the occurrence of PD in *Fritschiella*, but we could only access McBride 1967 [154], wherein they did not describe PD. ^e^ *Ulothrix fimbriata* not included since it is not officially reclassified yet (^a^ and Figure S3). The number of extant taxa (+x) was adopted from algaebase.org (accessed on 29 July 2023) [110]. +x refers to the number of species not unambiguously identifiable (in original literature as sp.). Red color indicates data derived from publications we could not fully access. Information is based on abstracts, previews, and citations.

#### 4.4.2. Ulvophyceae

The taxa of the Ulvophyceae have recently been reclassified based on phylogenomic analyses suggesting, e.g., that the Bryopsidales are sister group to the Chlorophyceae and are only distantly related to the Ulvophyceae sensu stricto (Figure 2 and Figure S1) [112,113]. Notably, special care must be taken with respect to the topic of this review, since many ‘Ulvophyceae’ species, which were investigated for the occurrence of PD/PDLCs in the old literature, have meanwhile been assigned to other taxa (Table S1 and discussion below).

Four main body plans can be distinguished in the Ulvophyceae, which typically live in marine habitats (Figure 2) [155]. Exclusively mononucleate unicells which may form small clusters and/or sarcinoid coenobia (type 1) occur in five orders of the Ulvophyceae with particularly small numbers of species (Scotinosphaerales, Ignatiales, Oltmannsiellopsidales, Sykidiales, and Chlorocystidales) [1,3,113]. Occurrence of PD has not been investigated in any of these orders, but in view of the simple body plans of the algae, the presence of PD would be unlikely. Other orders of the Ulvophyceae comprise multicellular algae composed of mononucleate cells (type 2) which might be filamentous (Trentepohliales, Ulotrichales, Ulvales) and form more complex sheets or tubes in the green sea lettuces (Ulvales). For *Ulva mutabilis*, however, it was reported that complex morphological structures are formed in the presence of particular bacterial strains only, and are lost in sterile cultures. This indicates the significance of cross-kingdom interactions for the establishment of complex multicellular body plans in the Ulvales [156,157,158]. Siphonocladous filamentous Ulvophyceae composed of multinucleate cells represent the next type of body plan (type 3) (Cladophorales, Acrosiphoniales including *Urospora* [112]). Yet, the most striking body plan of the Ulvophyceae is siphonous (type 4) and consists of a giant single cell (Dasycladales and Bryopsidales) that forms a macroscopic alga with morphologically and functionally differentiated regions termed ‘pseudo-organs’ [1]. The giant cells of Bryopsidales are always multinucleate, while many Dasycladales, e.g., the well-known *Acetabularia*, stay in a uninucleate stage with a large diploid macronucleus for a long time and multiple nuclear divisions only take place after meiosis in the course of sexual reproduction.

The siphonous (and siphonocladous) algae among the Ulvophyceae illustrate that, besides true multicellularity, there must be another way to achieve morphological complexity. Remarkably, differential gene expression was proven for different ‘pseudo-organ’ regions of siphonous *Caulerpa* cells (Bryopsidales) which might allow distinct morphological and functional differentiation in spatially separated cytoplasmic domains formed in the absence of cellular borders [1,159,160]. Given the intense cytoplasmic streaming in the Bryopsidales, however, those domains might not be permanently stable [161]. Yet, formation of nucleo-cytoplasmic domains with higher stability can be expected to occur in the siphonocladous Cladophorales and also in reproducing siphonous *Acetabularia* (Dasycladales). Their cell(s) share the common feature that nuclei are regularly spaced and are fixed in a particular domain of the stationary cytoplasm via microtubules [161,162,163]. In *Valonia ventriculosa* (Cladophorales), uninucleate domains are separated from each other by a system of interconnected vacuoles filled with a viscous mucilage leaving only thin, tonoplast-lined, interconnecting cytoplasmic strands (80–100 nm) between the domains (Figure 1T) [57].

Except for one study reporting the occurrence of non-PD cell connections in (fragmented?) *Codium* (see Section 2.4, Figure 1M,N, Table S1) [50], there are no reports on the occurrence of cell connections in any of the Ulvophyceae orders with siphonous or siphonocladous body plans, although cell division by furrowing was investigated, e.g., for the siphonocladous *Cladophora* [164]. Lack of PD in the multinucleate Ulvophyceae algae is, however, not well supported. Yet, supposed that multinucleate algae possibly represent intermediate stages on the evolutionary trajectory from unicellular—via siphonous—to multicellular body plans [4,5,9,155], the selection pressure for symplasmic intercellular communication might not be strong in the siphonous and siphonocladous state. On the contrary, it can be assumed that there might be a particular need to establish continuous cell walls as permanent symplasmic barriers between isolated domains, which then might communicate by other means than via symplasmic cell connections (e.g., ligand-receptor-mediated pathways). Even more complex multicellular body plans with initial cell-type differentiation might be established in this way as observed with the Cladophorales. This hypothesis requires experimental proof, though.

With respect to the occurrence of PD/PDLCs, the remaining orders of multicellular Ulvophyceae, which are composed of mononucleate cells, fall in two categories (i.e., with and without PD/PDLCs), which suggests an independent gain of multicellularity [155,165]. For the Ulvales, occurrence of PD/PDLC structures was not reported, although several investigations were performed on eight species from five distinct genera classified in four different families (Table 1 and Table S1). Thus, lack of PD in the Ulvales, which divide by furrowing [166], is well supported, but would not have been expected in view of their comparably complex body plans. However, the absence of PD would match the findings that (i) (complex) multicellularity in Ulva strongly depends on bacterial interaction [156] and (ii) the genes of the retinoblastoma (RB)/E2F pathway and associated D-type cyclins, known to mediate multicellularity and symplasmic connectivity in Volvocales (see Section 2.5 [99]), lack in the genome of *Ulva mutabilis* [158]. The presence of the respective genes in *Caulerpa* underlines the sister relationship between Bryopsidales and Chlorophyceae [158]. Yet, non-PD cell connections (‘septal plugs’) have been reported for *Ctenocladus circinnatus* (Ulvales) [49,89] (see Section 2.4).

Massive taxonomic reclassification was required in the Ulotrichales, since this taxon was intermingled with other filamentous green algae which, at present, are classified as Klebsormidiophyceae (*Klebsormidium*, *Interfilum* [167,168]) and Uronemataceae (*Uronema* species which were previously classified in the genus *Ulothrix* [169]). Data on the presence of cell connections in the Ulotrichales were drastically affected by these changes. In contrast to previous reviews [80,100,101,102], evidence for the occurrence of PDLCs in Ulotrichales was only found in one out of fourteen species from four distinct genera classified in four different families (Table 1 and Table S1). Since, remarkably, ‘*Ulothrix fimbriata*’ is the only PD/PDLC-bearing species within the eleven species of the genus *Ulothrix* investigated, we revisited its taxonomic position in a molecular phylogenetic tree based on 18S rRNA genes (Figure S3). In 99% of the generated trees, ‘*Ulothrix fimbriata*’ clusters among the genus *Uronema*, which also holds true for *Uronema gigas* whose taxonomic classification is not consistently treated at present. Based on this finding, we would conclude that ‘*Ulothrix fimbriata*’ should be reclassified as ‘*Uronema fimbriata*’, which would then result in a well-supported lack of PD/PDLC structures in the Ulotrichales as shown before for the Ulvales. This matches with the comparably simple body plans of the Ulotrichales, as they form uniseriate, rarely multiseriate, filaments which may show basal–apical polarity.

It is worth mentioning that cell division in Ulvophyceae is not always achieved by furrowing, as stated in the original description [77,170] and in many textbooks [151]. For some Ulotrichales, hybrid modes of cytokinesis were observed, which combine ingressive furrowing with the formation of a cellplate-like structure of linearly aligned (Golgi)vesicles [169], as described in Section 2.5 for *Volvox carteri* [51]. Nevertheless, formation of PD/PDLCs amongst the vesicles does obviously not occur in Ulotrichales, which indicates the lack of a particular component required for PD/PDLC formation in this taxon. Absence of a phycoplast in Ulvophyceae might be a decisive factor, but it would also be interesting to see whether Ulotrichales lack the genes of the retinoblastoma pathway as it was reported for the Ulvales [158].

In contrast to all other orders of the Ulvophyceae, however, occurrence of PDLCs was observed with each of the sixteen investigated species from three distinct genera classified in the single family Trentepohliaceae (Trentepohliales; Figure 2, Table 1 and Table S1). Trentepohliales are unique with respect to their exclusively terrestrial habitats where they are free living, occur as photobionts in lichens, or are associated with plants as epiphytes and endophytes [115]. This might have put a strong selection pressure towards the development of complex multicellularity and division of labor on this taxon. Moreover, the mode of cytokinesis is very special, since a phragmoplast-like structure [171,172] is involved in the formation of a cell plate that grows in centripetal direction and is subsequently attached to the parental cell wall. Remarkably, PCR amplifications even suggest the occurrence of phragmoplastin in Trentepohliales, a dynein-like protein mediating vesicle fusion during cell-plate formation in plants [173]. It is tempting to speculate whether this finding somehow correlates with the epi- and endophytic lifestyle of the algae and possibly hints at horizontal gene transfer. Yet, it is unknown whether there is a mechanism for phragmoplast guidance [174] involved in the anchorage of the cell plate in Trentepohliales and how this might be achieved in the absence of a preprophase band. In some species, a slight additional furrowing—and, thus, a hybrid mode of cytokinesis—was observed, while furrowing lacks completely in other Trentepohliales [172,175]. PDLCs of Trentepohliales (Figure 1S), which have a diameter of 40–50 nm and lack a central ER-component or other substructures in the cytosolic channel [175], are often aggregated in the median region of the cell wall to form a central pit [172]. Yet, the distribution of PDLCs might depend on the culture conditions and, thus, seems to be affected by environmental factors (Table S1 [49]).

#### 4.4.3. Chlorophyceae

The third major class of the core Chlorophyta are the Chlorophyceae which contain ecologically and morphologically diverse algae and split into two clades (Figure 2 and Figure S1) [1,3,176,177]. The OCC clade contains the Oedogoniales, Chaetopeltidales, and Chaetophorales, while the CS clade is formed by the Sphaeropleales and the Chlamydomonadales, which include the already described Volvocaceae (see Section 2.5). The sister group Sphaeropleales comprises a huge variety of common planktonic freshwater algae, e.g., the well-known water net *Hydrodictyon*, which show only simple body plans ranging from unicells to small clusters or filamentous forms composed of uninucleated or, notably, also multinucleated cells (Figure 2) [151]. Different modes of cell division have been described even for closely related species varying from simple furrowing, via hybrid modes, to an exclusively cellplate-mediated cytokinesis (Table S1). Lack of a phycoplast, which otherwise is regarded as a typical feature of the Phaeophyceae, was described for *Cylindrocapsa geminella* [178]. *Microspora* exhibits a particular mode of cell division that largely resembles the cytokinesis of Oedogoniales described in the next paragraph [151,179]. Data on the occurrence of cell connections in the Sphaeropleales is scarce, but no PD/PDLCs were observed in three genera classified in two different families (Table 1 and Table S1). Further investigations might be useful to confirm these data.

The Oedogoniales are unbranched or branched filamentous freshwater algae with specialized cell types like holdfasts and hair cells, and with a special mode of intercalary cell division [180]. Before the onset of mitosis, a membrane-lined ring filled with wall material is deposited peripherally at the apical end of the cell. After karyokinesis, an arrangement of aligned (Golgi)vesicles (the future cell plate) is formed by means of a phycoplast and, during subsequent growth of the daughter cells, the elongated parental longitudinal wall splits exactly in the position where the ring has been deposited before. Wall material released from the ring seals the split immediately. The aligned (Golgi)vesicles then move collectively towards the sealed split, and fuse more or less synchronously with each other to form a cell plate which is finally attached to the sealed parental wall [181]. It is unclear yet how the movement and the final position of the cell plate are controlled.

PD/PDLCs have been found in each of the two investigated genera classified in the single family Oedogoniaceae (Oedogoniales, Table 1 and Table S1). It would be interesting to receive further data for the third species-rich genus in this taxon, i.e., *Oedocladium*, as well as more detailed studies on the development of their cell connections, since the PDLCs of the Oedogoniales have a unique structure that differs significantly from all the other cell connections discussed before (Figure 1U). In *Bulbochaete hiloensis* [58], PDLCs are stable, wide, membrane-lined pores with an outer diameter of 86 nm which are surrounded by a cell-wall collar 120 nm in diameter. The inner face of the plasma membrane is decorated with helically arranged globular particles, reminiscent of the plasma membrane-associated particles observed in the cytosolic sleeve of land-plant PD [32,34] and resembling the membrane coatings found in cytoplasmic bridges of *Volvox* (see Section 2.5 [51]). A stabilizing function is attributed to the particles which leave an inner cytosolic lumen of 40–45 nm free. At the orifices, cell-wall collars form neck constrictions which limit the inner diameter to 8–15 nm. A central ER-derived substructure is lacking in these cell connections. Most strikingly, however, an electron-dense band crosses the PDLC at the level of the median plane of the cell wall. It is not known whether this plugging material restricts the SEL of the PDLC or occludes them entirely. The unique structure of the PDLCs in the Oedogoniales suggests a separate evolutionary origin.

The Chaetopeltidales comprise only sixteen species of unicellular freshwater algae and simple multicellular organism with filamentous and sheet-like thalli. The absence of PD in this taxon is a typical character that is already mentioned in the description of the order [182]. Consequently, there are only few studies on the occurrence of PDLCs in this group, but PDLCs have not been observed in any of the three analyzed genera of the family Chaetopeltidaceae (Table 1 and Table S1).

Contrary to the Chaetopeltidales, PDLCs have been proven to occur in many species of the direct sister group Chaetophorales that comprises unbranched or branched filaments with initial cell-type differentiation. Despite the taxonomic uncertainties which still exist within this group on the family level [111], PDLCs were observed in every family of the Chaetophorales, except for the recently described Barrancaceae [183] which comprise only three species and have only once been examined by TEM to date [184]. The available dataset comprises the analyses of twenty-five species from thirteen distinct genera classified in six different families (Table 1 and Table S1).

PDLCs in Chaetophorales develop during typical Chlorophyceae cytokinesis, which is achieved by means of a cell plate and an associated phycoplast (Table S1) [185]. Their diameters resemble that of canonical land-plant PD [100], but a central ER-derived (sub)structure is lacking in most species (Figure 1S). However, as described for some fungal species, a solid ‘core’ (*Stigeoclonium helveticum* and ‘*Ulothrix fimbriata*’ = *Uronema fimbriata*, Figure 1R and Figure S3 [53]) or even an ER-associated ‘desmotubule’ (*Aphanochaete elegans*, Figure 1A [185]) was sporadically observed to traverse (predominantly young) PD/PDLCs in a few Chaetophorales species. However, these observations were called into question by other authors who did not find such ER substructures in mature PD (*Aphanochaetae* [100]). It might be possible, however, that the ER of some Chaetophorales is only transiently associated with the PDLCs and differs from true desmotubules which represent modified ER-membrane structures persisting as permanent constituents in canonical PD [32,34].

Another remarkable exception within the Chaetophorales is *Sporocladopsis novae-zelandiae* for which a cell division by centripetal infurrowing has been described and no contribution of vesicles was mentioned [186]. Nevertheless, PDLCs with a diameter of 40 nm were observed in these infurrowing walls and it was speculated whether they may have formed by membrane invagination and protrusion just like PDLCs in Phaeophyceae (see Section 4.2). Interestingly, *Sporocladopsis novae-zelandiae* is a highly specific epiphyte of two *Lessonia* species (Laminariales/Phaeophyceae) [187] and, again, it can be speculated that there might possibly be a significant correlation between these observations which hints at horizontal gene transfer. It might be worth considering this particular species in future studies, also with respect to its taxonomic classification within the Chaetophorales.

### 4.5. PD/PDLCs Structures in the Green Lineage—Viridiplantae (Streptophyte Algae)

The Streptophyta are the direct sister group of the Chlorophyta (Figure 2 and Figure S1). They comprise the streptophyte algae, which typically have freshwater or—less frequently—moist terrestrial habitats, and the land plants (Embryophyta) which have undergone the water-to-land transition and conquered land some 500 MYA [188]. The streptophyte algae are subdivided in two grades. The KCM grade contains the two sister groups Mesostigmatophyceae and Chlorokybophyceae besides the Klebsormidiophyceae, while the ZCC grade includes the Charophyceae, Coleochaetophyceae, and Zygnematophyceae.

As might have been expected, there are no reports for the occurrence of PD in the two streptophyte algal classes which are positioned at the base of radiation as sister to all other Streptophyta—neither for the Mesostigmatophyceae [189] whose members are scaly flagellate unicells in freshwater habitats resembling the ‘prasinophyte’ classes in the chlorophytes phylum, nor for the Chlorokybophyceae (Table 2) [108,190] which comprise members with sarcinoid body plans in moist terrestrial habitats [1,3,114]. PD/PDLCs also lack in the Klebsormidiophyceae living as single cells, sarcinoid coenobia, or unbranched, filamentous algae which divide by furrowing [191], but the formation of pseudofilaments and packet-like cell clusters resulting from sporulation-like cell divisions has also been discussed [179]. PD/PDLCs were not observed in eighteen distinct species of two genera of the Klebsormidiaceae (Table 2 and Table S1, *Interfilum* was included according to [167,168]).

**Table 2:** Streptophyte algae species which have been studied for PD/PDLC presence sorted by order, family, and genus. Numbers of examined species per genus, numbers of PD/PDLC-containing species, and numbers of relevant publications are shown. Based on Table S1.

Class	Order	Family	Genus	n (Species Examined)		n (Publications)
				Total	with PD (LC)	
Chlorokybophyceae	Chlorokybales	Chlorokybaceae	*Chlorokybus*	1	0	1
Klebsormidiophyceae	Klebsormidiales	Klebsormidiaceae	*Klebsormidium*	10 + 2	0	6
			*Interfilum* ^a^	3 + 3	0	2
			+4			
		+1				
Charophyceae	Charales	Characeae	*Chara*	6 + 2	6 + 2	13
			*Nitella*	2	2	3
			+48			
		+5				
	+3					
Coleochaetophyceae	Coleochaetales	Coleochaetaceae	*Coleochaete*	5	5	5
			+4			
	+1					
Zygnematophyceae	Spirogyrales	Spirogyraceae	*Spirogyra*	1	0	1
			+1			
	Zygnematales	Zygnemataceae	*Zygnema*	0 + 3	0	1
			*Mougeotia*	2 + 1	0	2
			+24			
		+1				
	+3					

The number of extant taxa (+x) was adopted from algaebase.org (accessed on 29 July 2023) [110]. ^a^ *Interfilum* was included according to [167,168]. +x refers to the number of species not unambiguously identifiable (in original literature as sp.).

**Figure 3:**
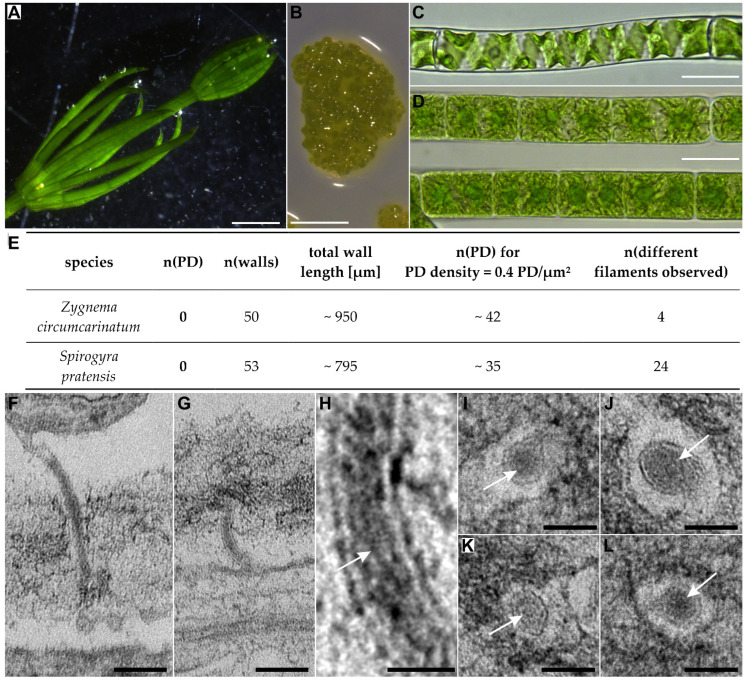
Light microscopical images of the investigated streptophyte algal species (**A**) *Chara fragilis* (Charophyceae), (**B**) *Coleochaete scutata* (Coleochaetophyceae), (**C**) *Spirogyra pratensis*, and (**D**) *Zygnema circumcarinatum* (both Zygnematophyceae). (**E**) Data obtained from TEM investigation on *Z. circumcarinatum* and *S. pratensis*. Numbers of algal filaments, cell walls, and total cell-wall lengths examined without discovering any PD/PDLCs. For reference, the theoretical value of expected PD/PDLCs is given, which is based on the lowest reported PD density (0.4 PD/µm^2^) in [200] and the equation of Gunning [210]. (**F**–**H**) Longitudinal and (**I**–**L**) cross-sectional views of PDLCs of *C. scutata* observed by TEM. (**H**) shows a detail of (**G**) with higher contrast to emphasize the inner, central structure (arrow). Inner structures can also be identified in the cross sections (arrows). Images and data taken from [199]. scale bars: (**A**) 2 mm; (**B**) 200 µm; (**C**,**D**) 25 µm; (**F**,**G**) 100 nm; (**H**) 20 nm; (**I**–**L**) 50 nm.

**Figure 4:**
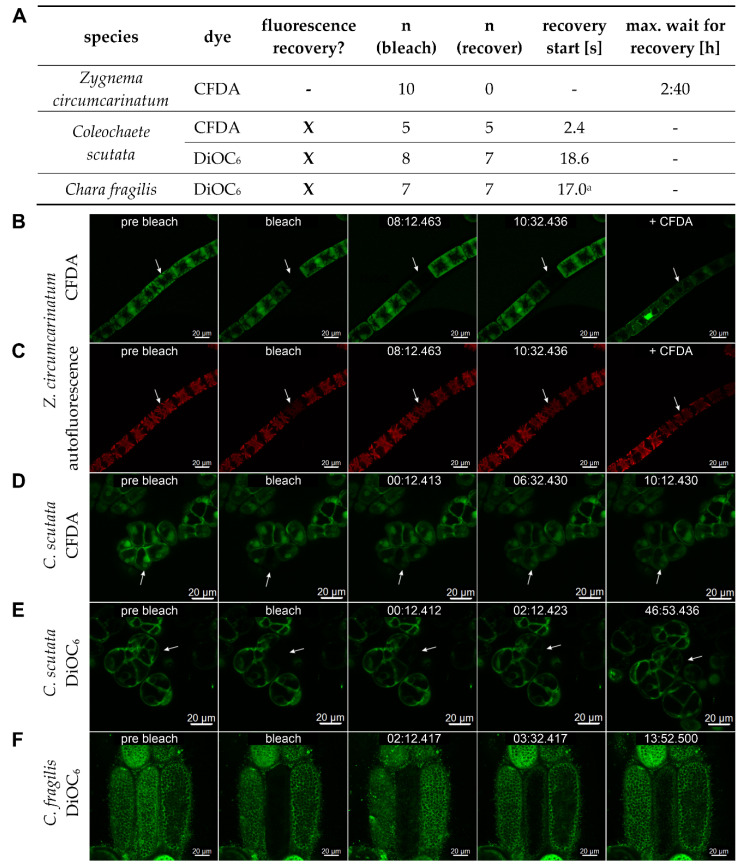
Fluorescence recovery after photobleaching (FRAP) experiments for the streptophyte algae *Zygnema circumcarinatum* (Zygnematophyceae), *Coleochaete scutata* (Coleochaetophyceae), and *Chara fragilis* (Charophyceae). (**A**) Summary showing numbers of successfully bleached cells for different fluorescent dyes, numbers of cells recovering fluorescence, average time until onset of recovery was observed [s], and maximal time without observance of fluorescence recovery after bleaching [h]. (**B**) Exemplary experiment on *Z. circumcarinatum* without fluorescence recovery of the cytosolic marker CFDA. To assure the survival of the bleached cells, CFDA—which shows fluorescence only after entering living cells and transformation to CF—was reapplied 25 min post bleach (last panel). (**C**) As a second control to guarantee cell survival, autofluorescence of the chloroplasts was monitored which respond sensitive to high irradiation. In *C. scutate*, both, (**D**) the cytosolic CFDA and (**E**) the ER-binding DiOC_6_ fluorescence was quickly recovered. (**F**) Similarly, *C. fragilis* cortex cells recovered DiOC_6_ fluorescence. Images and data taken from [199]. ^a^ One FRAP experiment was excluded from the average calculation since the first image was recorded after 03:32:427 when the recovery process was already in progress. All scale bars 20 µm.

The remaining three classes of ZCC streptophyte algae, together with the land plants, share the common feature that a phragmoplast is present during cell division although other characters of cytokinesis vary significantly between the taxa [174]. The Charales (Charophyceae) and the Coleochaetales (Coleochaetophyceae) have developed the most complex body plans and, for a certain period, each group was considered to represent the closest algal relative of land plants, which however turned out to be wrong [192,193]. The macroscopic *Chara* algae (Figure 3A) [194], known as stoneworts, grow with apical cells to form complex three-dimensional thalli composed of rhizoids and erected branched axes, differentiated in multicellular nodes and giant multinucleate internodal cells which might be corticated. Depending on the species, Coleochaetales form branched filaments or two-dimensional disc-shaped pseudoparenchymatous or parenchymatous thalli growing with apical cells at the margins [195,196] (Figure 3B). Remarkably, the morphologically less elaborate Zygnematophyceae which live as unicells, small cell clusters, or unbranched filaments without cell differentiation are the direct extant sister group of land plants (Figure 3C,D) [7,192,193]. This surprising finding was recently explained by the evolutionary scenario that the body plan of the most recent ancestor of the Zygnematophyceae was reduced to an unicellular state, and simple multicellularity evolved de novo five times in independent lineages of this taxon [7]. Zygnematophyceae divide by furrowing or, as shown for *Spirogyra* and *Mougeotia*, by a ‘hybrid mode’ of division [174,197]. Consistent with their very simple body plans, Zygnematophyceae have often been reported to lack PD. However, in our analysis, we could only find four studies which actually deal with the presence of PD/PDLCs in three different genera of two distinct families classified in two different families (Table 2 and Table S1), although Zygnematophyceae have been investigated in many recent ultrastructural studies, e.g., [198]. Thus, we decided to verify the data in a targeted transmission electron microscopy study searching for PD in *Zygnema circumcarinata* and *Spirogyra pratense*, representing two major Zygnematophyceae orders (Figure 3E taken from [199]). We did not observed any PD/PDLCs in both species, although the number of interfaces analyzed (n = 50 and n = 53, respectively) would assure that approx. 40 PD/PDLCs should have been found when extremely low, previously published PD-density values [200] were assumed to occur in the Zygnematophyceae. Moreover, in functional tests using Fluorescence Recovery After Photobleaching (FRAP), fluorescence of the cytosolic marker carboxyfluorescein(diacetate) (CFDA) was not restored via symplasmic import from neighboring cells in 100% of the experiments performed with *Z. circumcarinatum* (Figure 4A–C taken from [199]), which confirms the symplasmic isolation of adjacent cells.

In contrast, PD/PDLCs have been found in all analyzed species of the morphologically complex Charophyceae and Coleochaetophyceae, i.e., with ten species from two distinct genera classified in the Characeae and with five species of the genus *Coleochaete* in the Coleochaetaceae (Table 2 and Table S1).

For dividing cells at the margin of the disc-shaped *Coleochaete* thalli, different modes of cytokinesis have been described to occur with radial or circumferential cell divisions (i.e., perpendicular or parallel to the thallus surface [201,202]). Later, it was shown, however, that cell divisions in both planes are polarized with an acentric starting point for phragmoplast-driven cell-plate formation [203]. Simple, narrow PD/PDLCs with a diameter of approx. 25 nm develop in the new cell walls, but the mechanism of formation has not been demonstrated yet. So far, no central substructures have been reported for the PD/PDLCs of Coleochaetophyceae [100].

For the complex *Chara* thalli, cytokinesis is uniformly achieved by means of phragmoplast-mediated cell-plate growth [204], but reports on the mode of PD/PDLC formation and the (sub)structure of the PD/PDLCs vary drastically. Primary formation of PD/PDLCs among the fusing vesicles of the cell plate was usually reported [204], whereas only secondary PD/PDLC formation was found in another *Chara* species [205]. Thus, there is still no final answer on the mode of PD formation in this genus. Furthermore, there are varying reports on the presence/absence of an internal PD/PDLC structure, which might be associated/not associated with the ER, and is occasionally attached to the plasma membrane via spoke-like connections as in canonical type II PD (Figure 1A,B,W) [34,59,204,205,206]. One scenario to explain this variability would probably be that there is only a transient association of the ER and the PD/PDLCs in many *Chara* species [59,207], but species-specific differences might also be considered which would diversify PDLCs without or with only a transient central ER component and PD with a permanent ER component. Remarkably and unique among algal PD/PDLCs, besides simple PD/PDLCs, complex branched cell connections have often been found in older *Chara* walls, indicating that morphological modifications of the PD/PDLC take place during development, as was observed with angiosperms (Figure 1C, with or without desmotubules). Moreover, a large size-exclusion limit of 45 kDa was reported for the PD/PDLCs at the interface between nodal and internodal cells of *Nitella*, a genus of the Charales [208], and electrical coupling was also proven [209].

Detailed information on the PD/PDLCs in the ZCC algal grade is particularly relevant, because it has been suggested that land-plant PD originate from the same evolutionary line of the monophyletic phragmoplastophyta [80,104,109,211]. This would imply homology of all streptophyte PD and a secondary loss of PD connectivity in the Zygnematophyceae, which is supported by the recent findings on the loss and re-gain of multicellularity in this taxon [7]. Alternatively, the evolution of streptophyte PD might have occurred independently in Charophyceae/Coleochaetophyceae and in the land plants as a concomitant phenomenon accompanying the multiple evolution of parenchyma-like, multicellular tissues. The latter scenario is favored by several PD researchers [103,118,212] due to the following reasons: (i) There are uncertainties about the homology of the molecular components in the cell connections of streptophyte algae and land plants. Information is scarce, but in *Chara corallina* cell fractions prepared from PD-rich nodal regions, only two of the already-known, conventionally identified PD proteins were found by 2-D gel electrophoresis, peptide mass fingerprinting, and LC-tandem mass spectrometry analyses [213]. This might either point to a separate evolutionary origin between the two types of cell connections, or might simply reflect the large evolutionary distance between their PD/PDLC proteins. Notably, even within land-plant PD, protein components do not seem to be highly conserved, since the typical DUF-26 domain-containing PD-localized proteins (PDLPs) used as markers for the PD of eudicots were not found in the bryophyte model plants *Marchantia polymorpha* and *Phycomitrella patens* [214]. (ii) Uncertainties about the occurrence of a desmotubule-like ER component within the PD/PDLCs of streptophyte algae is another major aspect that casts doubt on the homology with land-plant PD. The rationale behind it is that the occurrence of a permanent ER component would render a homology with land-plant PD more likely, because it does not occur in PDLCs of any other algal group, and transient ER associations were also only rarely observed with PDLCs of Chaetophorales and in the large, special PDLCs of the Volvocaceae. We tried to address this point with transmission electron microscopy of *Coleochaete scutata* (Figure 3F–L taken from [199]) and could regularly find a central component with a rod-like core within the PDLC, but an association with the cytoplasmic ER was not found (Figure 1V). However, when we performed FRAP experiments on *C. scutata*, we could not only observe the fast recovery of CF(DA) fluorescence in 100% of the experiments performed (Figure 4A,D), but also the recovery of the fluorescence signal of DiOC_6,_—a lipophilic cation dye used for ER staining—in seven out of eight experiments (Figure 4A,E taken from [199]). This indicates not only that the cytosol of neighboring *Coleochaete* cells is interconnected, but that their ER systems are too. Similar results were obtained with *Chara fragilis* cortex cells, which showed an even faster recovery of DiOC_6_ fluorescence in 100% of our FRAP experiments (Figure 4A,F taken from [199]). Being aware that DiOC_6_ does not stain the ER in a highly specific manner [215], we tried to repeat our experiments with ER-Tracker Green (BODIPY FL Glibenclamide, ThermoFisher Scientific, Dreieich, Germany), but this dye did not stain the algal cells [199,216]. However, the data presented hint at a continuous ER-membrane connectivity between neighboring algal cells of *Coleochaete scutata* and *Chara fragilis* which is likely mediated by the PD as observed with land-plant PD. It is possible that, in the streptophyte algae, the association of the cytosolic ER to the central ER component of the PD is less firm than in land-plant PD and, thus, is often lost during the electron microscope preparation processes, which underlines the significance of functional tests.

## 5. Conclusions II

(1) The data gathered from the literature to map the present-day distribution of PD/PDLCs on today’s taxonomic classification of extant algal lineages (Figure 2, Figures S1 and S2) show that there are several taxa which have not or not sufficiently been investigated, although they have an interesting phylogenetic position; e.g., the Discosporangiales at the base of the radiation of Phaeophyceae, the newly described Barrancaceae within the Chaetophorales, the Acrosiphoniales and Cladophorales within the Ulvophyceae, the genus Oedocladium in the Oedogoniales, and, of course, the streptophyte algae Coleochaetales and Charales.
(2) According to the available data, we would conclude, however, that there have been at least six independent origins of PD/PDLCs:
  - at least one in the Phaeophyceae, probably before the start of their radiation;
  - an extraordinary one in the Trentepohliales, which, after the proposed reclassification of ‘*Ulothrix fimbriata*’ as ‘*Uronema fimbriata*’, would be the only PDLC-bearing taxon in the Ulvophyceae. The evolution of PDLCs in the Trentepohliales might be a consequence of a special selection pressure in their terrestrial habitats or a prerequisite for the survival in their ecological niche (see Section 4.4.2);
  - most likely three in the Chlorophyceae:
    (i) Volvophyceae (Chlamydomonadales, CS clade), since their ‘cytoplasmic bridges’ resemble PDLCs rather than non-PD cell connections if the mechanism of their formation is considered;
    (ii) Oedogoniales whose PDLCs differ structurally from all other algal PDLCs;
    (iii) Chaetophorales (possibly with a very special situation in *Sporocladopsis*; see Section 4.4.3);
  - at least one in the Phragmoplastophyta, including the land plants. Homology of all PD in this taxon would be supported by the presented hints on the occurrence of a permanent ER substructure in their PD. Yet, two or three independent origins of PD/PDLCs in the Phragmoplastophyta can still not be ruled out.
(3) Data must be considered with care, though, since the present-day occurrence of PD/PDLCs in extant algal taxa might not mirror the complete picture of PD evolution. In view of the enormous age of the green lineage, it can be expected that several independent transitions of unicellular green algae to coenocytic, siphonous, and multicellular body plans with increasing complexity have taken place. However, reductions of algal body plans have also occurred in other lineages [7] so that complexity and even multicellularity have been lost at several evolutionary points. This interplay between gain and loss of traits hampers inferences about the evolution of particular characteristics, like the occurrence of PD.
  Moreover, as discussed in Section 4.1 (fungi), Section 4.4.2 (Ulvales, Trentepohliales), and Section 4.4.3 (*Sporocladopsis novae-zelandiae*), it should be taken into account that other mechanisms like horizontal gene transfer and cross-kingdom interactions might have influenced the final picture [157,217,218]. It will be a challenging task for future studies to decipher the impact of symbiotic interactions between eukaryotes and bacteria or viruses, as well as between different eukaryotic lineages on the evolution of multicellularity and symplasmic cell connections.
(4) According to the data presented, PD/PDLCs have evolved in parallel with complex multicellularity rather than with simple multicellularity [19]. Supposedly, direct symplasmic communication was selected for as a group-level trait [23,24] correlated with the evolution of division of labor. It might be a prerequisite for—or a direct consequence of—coordinated development and cooperative functioning of distinct cell types within an algal thallus, in particular with those who had probably undergone the transition from unicellular—via colonial—to multicellular body plans [4,5]. Throughout the phyla, simple multicellular algal organisms were found to lack both cell-type diversification and cell connectivity (i.e., Xanthophyceae, Trebouxiouphyceae, Ulotrichales, Chaetopeltidales, Klebsormidiophyceae, Zygnematophyceae; Figure 2 and Figure S2), while complex multicellular body plans with cell-type specification often coincide with the occurrence of PD/PDLCs (PD-bearing Phaeophyceae, Trentepohliales, Volvocaceae, Oedogoniales, Chaetophorales, Charophyceae, Coleochaetophyceae, and Embryophyta; Figure 2 and Figure S2).
  The only exceptions would be the following:
  (i) the Ulvales whose complex body plans presumably have evolved in the absence of PD/PDLCs via a distinct pathway dependent on cross-kingdom interaction with bacteria;
  (ii) the siphonocladous Cladophorales and the (partially siphonocladous) Sphaeropleales which also show complex multicellularity, but lack PD/PDLCs. Independent loss of PD/PDLCs in the latter two taxa might be a conceivable explanation for this finding, but, as speculated in Section 4.4.2, it is also possible that these taxa have achieved complex multicellularity via the ‘siphonous–multicellular’ transformation [4,5] and presence of specialized structures for symplasmic communication might not be a decisive factor in this evolutionary pathway.
(5) We suggest to differentiate clearly between distinct types of cell connections, which can be assumed to have distinct functional capacities (Figure 1):
  - Membrane-lined cytosolic channels, enabling plasma-membrane connectivity besides cytosolic connectivity, should be distinguished from other types of cell connections including the proteinaceous gap junctions of animals.
  - Cytoplasmic pores developing as single, large channels after incomplete abscission in a furrowing process (and functionally regulated by plugging) should be distinguished from multiple smaller PD/PDLCs which develop during cytokinesis in a cell plate-like structure (either among the fusing vesicles or by protrusion) or which are formed secondarily in a rigid cell wall.
  - PDLCs representing the dominant type(s) of symplasmic connections throughout the algal taxa should be distinguished from canonical PD which contain a permanent ER component and, thus, provide connectivity of the endomembrane systems of neighboring cells.
(6) Based on the presented data, we suggest a four-step process towards the evolution of canonical PD:
  (i) Establishment of specific plasma-membrane domains [219,220,221] is most likely necessary to control the membrane stability/flexibility required to form small cytosolic channels with relatively constant inner diameters of about 20 nm (reminiscent of Type I PD [34]) in a cell-plate(-like) structure during cytokinesis. This may happen either by leaving open pores between the fusing vesicles or by membrane invaginations (Phaeophyceae) into the developing wall. The latter would pave the way for secondary PD/PDLC formation as found with Phaeophyceae and angiosperms. It is worth noting that PDLCs may develop in centripetally (many algae) or centrifugally growing cell plates in which vesicle fusion occurs either simultaneously (many algae) or successively. The ER is obviously not necessarily required for this process, but a cell plate alone would probably not be sufficient to produce PDLCs as shown with the Ulotrichales (see Section 4.4.2). Membrane domains with proper lipid and protein equipment and, possibly, the presence of a phycoplast or a phragmoplast during cytokinesis might be indispensable for PD/PDLC formation.
  (ii) Recruitment of (proteinaceous) substructures attached to the inner face of the plasma-membrane lining the PDLC can be assumed to stabilize the PDLC, as shown with Oedogoniales and with Volvocaceae (Figure 1O,P,U).
  (iii) Targeted establishment of barriers within the cytosolic channel of the PDLCs as shown for Phaeophyceae, Oedogoniales, and some Chaetophorales presumably limits and controls the symplasmic transport capacity. Alternatively or in parallel, specialized cell-wall areas form collars around the PDLC (Phaeophyceae) or neck constrictions at the orifices (Oedogoniales) which may control the transport pathways functionally. Specialization of the cell-wall collars around PDLCs might also be a prerequisite for the development of PD twinning as a mode to form PD secondarily in existing walls (Phaeophyceae) and also for branching as a secondary modification of PD/PDLCs (*Chara*).
  (iv) Recruitment of the ER to the PDLC and a permanent fixation of the ER as indelible substructure of the cell connections gives rise to canonical PD enabling an additional endomembrane transport pathway. This process is likely mediated by special membrane-tether proteins and modifications of the ER membrane [124,125,126]. A temporary attachment of the ER to PDLCs, as suggested for some Chaetophorales and some *Chara* species, might represent an intermediate stage in this process.

## 6. Outlook

(1) Functional investigations on the symplasmic connectivity mediated by algal PD/PDLCs have only rarely been performed, and, thus, we know hardly anything about the size and biochemical nature of molecules which are transported through PD/PDLCs in Phaeophyceae, Chlorophyta, and streptophyte algae. We need to answer the following questions before we can conclude which role PD/PDLCs might play in the coordination of developmental processes of the algal thalli or in their cell- or non-cell-autonomous responses to environmental changes and (a) biotic stresses.
  (i) What is the size-exclusion limit for diffusional transport through algal PD/PDLCs?
  (ii) Is there also a targeted transport of macromolecules, like transcription factors, mRNA, and small RNA species with regulatory functions, as was reported for angiosperm PD?
  (iii) Is there a membrane flow through algal PD/PDLCs or is the intercellular exchange of lipids restricted by plasma-membrane domains like in angiosperm PD [221]?
  (iv) Are there differences in the numbers and/or functional capacities of PD/PDLCs at the diverse cell interfaces of the algal thalli which might establish distinct symplasmic domains like in angiosperm tissues?
  (v) Do algae alter their PD/PDLC numbers and/or the functional capacities of their symplasmic cell connections temporarily or permanently during development or in response to physiological stresses like angiosperms do?
  (vi) How are putative functional changes of algal PD/PDLC permeability and possible alterations of algal PD/PDLC frequencies achieved?
(2) Furthermore, we know almost nothing about the molecular composition of the algal PD/PDLCs in distinct taxa, except for the fact that typical molecular components of angiosperm PD lack in the streptophyte algae and only single potentially orthologous genes can be identified [222]. Proteomics on algal PDLCs has not been performed, except for a single initial study on *Chara corallina* [213]. Whether morphological similarities of PD/PDLC in closely or distantly related taxa reflect any homologies on the molecular level is unclear. Do PDLCs in distinct taxa differ with respect to their molecular PDLC equipment, and can the (respective) ‘molecular evolution’ of (analogous) PDLC be traced back with the help of recent species? Final conclusions on the evolution of PD/PDLCs cannot not be drawn without molecular data.

## 7. Material and Methods

### 7.1. Ulothrix fimbriata 18S rRNA Gene Phylogeny

Genomic sequences of 18S rRNA genes were retrieved from NCBI (*Ulothrix* sp. and *Uronema* sp.) and BLAST searches [223] using JGI Phytozome (*Volvox carteri*, *Chlamydomonas reinhardtii* and *Chromochloris zofingiensis*) [224] and NCBI (*Chara* sp.) [225]. Sequences were aligned using MAFFT (--localpair --maxiterate 10,000 --reorder) [226]. Phylogeny (Maximum Likelihood, 100 bootstraps) was generated using iqtree (-T AUTO -m TEST -b 100 -con) [227].

### 7.2. Algae Cultures

Algae cultures of *Zygnema circumcarinatum* (SAG 698-1b), *Spirogyra pratensis* (MZCH10213), and *Coleochaete scutata* (SAG 110.80M) were kindly provided by Prof. Dr. Jan de Vries from the Culture Collection of Algae at the University of Göttingen, Germany (SAG) within the MAdLand consortium. *Chara fragilis* was collected in the botanical garden of the Justus-Liebig-Universität Giessen (GPS 50°35′09.5″ N, 8°40′44.5″ E, from a pond) and identified by two dichotomous keys [228,229].

*Z. circumcarinatum* (ESP-medium (http://sagdb.uni-goettingen.de/culture_media/01%20Basal%20Medium.pdf (accessed on 14 September 2023))), *S. pratensis* (MiEB_12_-medium (http://sagdb.uni-goettingen.de/culture_media/07%20Desmidiacean%20Medium.pdf (accessed on 14 September 2023)), WC-medium (http://sagdb.uni-goettingen.de/culture_media/24%20WC%20Medium.pdf (accessed on 14 September 2023))), and *C. scutata* (WC-medium) were grown axenically in liquid cultures and on plates with solid medium (liquid media with 2% (*w*/*v*) agar). They were cultured under long-day conditions (16/8 h (70–90 µmol·m^−2^s^−1^)) at 22 °C. *C. fragilis* was freshly collected before experiments and stored in pond water for a maximum of two days under the aforementioned conditions.

Light microscopic images were taken with the Leica DM 5500 microscope B and Leica M165 C stereo microscope equipped with a Leica DFC 450 camera (Leica, Bensheim, Germany) using the Leica Application Suite (Version 4.3.0) software.

### 7.3. Transmission Electron Microscopy (TEM)

TEM experiments were performed as described in [230] with slight modifications for the fixatives. *Z. circumcarinatum* filaments were fixed on ice for 2.5 h in 2.5% glutardialdehyde (GA) in 20 mM cacodylate buffer (pH 7.2, adapted from [231]), *S. pratensis* filaments for 3 h in 2.5% GA in 50 mM cacodylate buffer (pH 7.2, adapted from [232]), and *C. scutata* thalli for 3 h in 2% GA in 25 mM cacodylate buffer (pH 7.2, adapted from [233]).

### 7.4. Fluorescence Recovery after Photobleaching (FRAP)

For the FRAP experiments, *Z. circumcarinatum, C. scutata*, and *C. fragilis* were stained with 1 mM CFDA ((5-(and-6)-Carboxyfluorescein Diacetate) or 2 µg/mL DiOC_6_(3)—diluted in ES-medium, WC-medium, or tap water, respectively—for 20 min at RT and washed 3 times in the respective medium/tap water. A Leica TCS SP8 confocal laser scanning microscope (Leica, Bensheim, Germany) was used for the microscopy with the Leica Application Suite X software utilized for image processing. CFDA and DiOC_6_ were excited with a 488 nm argon laser, and for chloroplast autofluorescence a 633 nm helium neon laser was used. Emission was detected at 507–557 nm and 660–690 nm, respectively, via PMT detectors at 63× magnification (HC PL APO CS2 63×/1.20 WATER immersion objective). For the FRAP experiments, the regions of interest were bleached at 50–80% laser power with 60–100% laser intensity for 10–80 frames (512 × 512 px, 400 Hz bidirectional scan, 2 AU pinhole, ‘Zoom In’ setting) with the 476, 488, and 496 nm laser lines of the argon laser active simultaneously.

## Supplementary Materials

The following supporting information can be downloaded at: https://www.mdpi.com/2223-7747/12/18/3342/s1, Figure S1: Phylogeny including the Phaeophyceae and Viridiplantae with focus on the occurrence of PD/PDLC; Figure S2: Phylogeny of the Stramenopila (especially Phaeophyceae) with focus on the occurrence of PDLC, body plans, and presence of cell differentiation; Figure S3: Midpoint rooted Maximum Likelihood phylogeny of 18S rRNA genes of *Uronema* and *Ulothrix* species; Table S1: List for the presence of PD/PDLC and non-PD cell connection in Viridiplantae. References [234,235,236,237,238,239,240,241,242,243,244,245,246,247,248,249,250,251,252,253,254,255,256,257,258,259,260,261,262,263,264,265,266,267,268,269,270,271,272,273,274,275,276,277,278,279,280,281,282,283,284,285,286,287,288] are cited in the Supplementary Materials.
