# Supplementary material for: Multicellularity and the Need for Communication—A Systematic Overview on (Algal) Plasmodesmata and Other Types of Symplasmic Cell Connections
Source: Plants (Basel). 2023 Sep 21;12(18):3342. doi: 10.3390/plants12183342 (PMC10536634; doi:10.3390/plants12183342)
Supplement: Supplementary file 1 [file plants-12-03342-s001.zip › plants-2559708-supplementary.pdf]

plants

MDPI

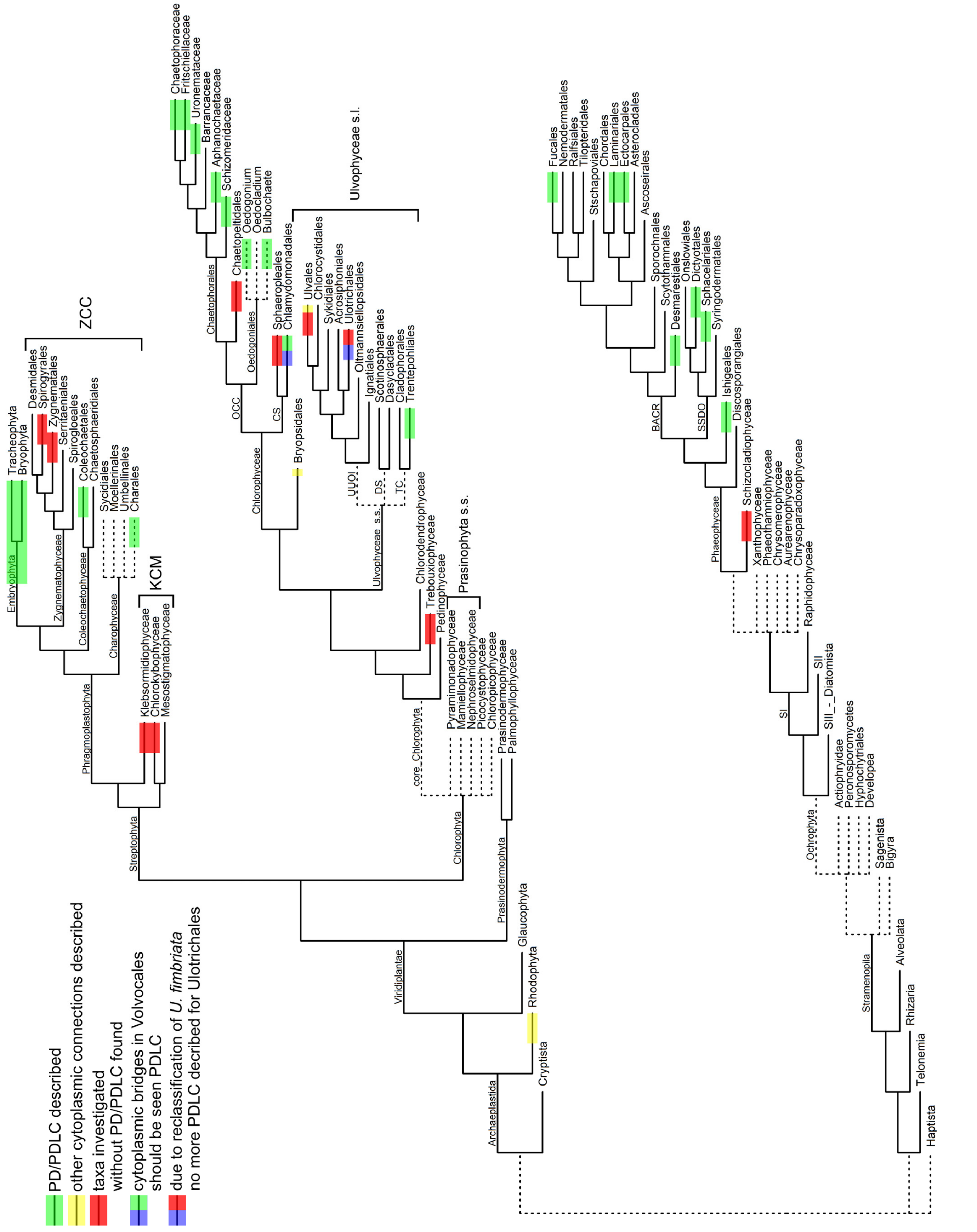
PD/PDLC described
other cytoplasmic connections described
taxa investigated without PD/PDLC found
cytoplasmic bridges in Volvocales should be seen PDLC
due to reclassification of U. fimbriata no more PDLC decribed for Ulotrichales
ZCC
KCM
Ulvophyceae s.l.
Prasinophyta s.s.
Tracheophyta
Bryophyta
Embryophyta
Desmidiales
Spirogyrales
Zygnematales
Serritaeniales
Spiroglocales
Colechaetales
Chaetosphaeridiales
Sycidiales
Moellerinales
Umbellinales
Charales
Zygnematophyceae
Coleochaetophyceae
Charophyceae
Phragmoplastophyta
Klebsormidiophyceae
Chlorokybophyceae
Mesostigmatophyceae
Streptophyta
Chaetophoraceae
Fritschiellaceae
Uronemataceae
Barrancaceae
Aphanochaetaceae
Schizomeridaceae
Chaetopeltidales
Oedogonium
Oedocladium
Bulbochaete
Chaetophorales
Oedogoniales
OCC
Sphaeropleales
Chlamydomonadales
CS
Chlorophyceae
Bryopsidales
Ulvales
Chlorocystidales
Sykidiales
Acrosiphoniales
Ulotrichales
Oltmannsiellopsidales
Ignatiales
Scotinosphaerales
Dasycladales
Cladophorales
Trentepohliales
UUOI
DS
TC
Ulvophyceae s.s.
Chlorodendrophyceae
Trebouxiophyceae
Pedinophyceae
core_Chlorophyta
Pyramimonadophyceae
Mamiellophyceae
Nephroselmidophyceae
Picocystophyceae
Chloropicophyceae
Prasinodermophyceae
Palmophyllophyceae
Chlorophyta
Prasinodermophyta
Viridiplantae
Glaucophyta
Rhodophyta
Cryptista
Archaeplastida
Fucales
Nemodermatales
Ralfsiales
Tilopteridales
Stschapoviales
Chordales
Laminariales
Ectocarpales
Asterocladales
Ascoseirales
Sporochnales
Scytothamnales
Desmarestiales
Onslowiales
Dictyotales
Sphacelariales
Syringodermatales
BACR
SSDO
Ishigeales
Discosporangiales
Phaeophyceae
Schizocladiophyceae
Xanthophyceae
Phaeothamniophyceae
Chrysomerophyceae
Aurearenophyceae
Chrysoparadoxophyceae
Raphidophyceae
SII
SIII_-_Diatomista
SI
Actiophryidae
Peronosporomycetes
Hyphochytriales
Developea
Ochrophyta
Sagenista
Bigyra
Stramenopila
Alveolata
Rhizaria
Telonemia
Haptista

**Figure S1:** Phylogeny including the Phaeophyceae and Viridiplantae with focus on the occurrence of plasmodesmata (PD)/ plasmodesmata-like cell connections (PDLCs). The phylogeny is based on [7,77,106-108,110-113,130]. The information on PD presence is based on Tables 1, 2, S1. Dotted branches indicate polytonies which are not sufficiently supported yet or groups whose exact relationship is not essential for the topic of this review. The branch lengths do not represent phylogenetic distances.

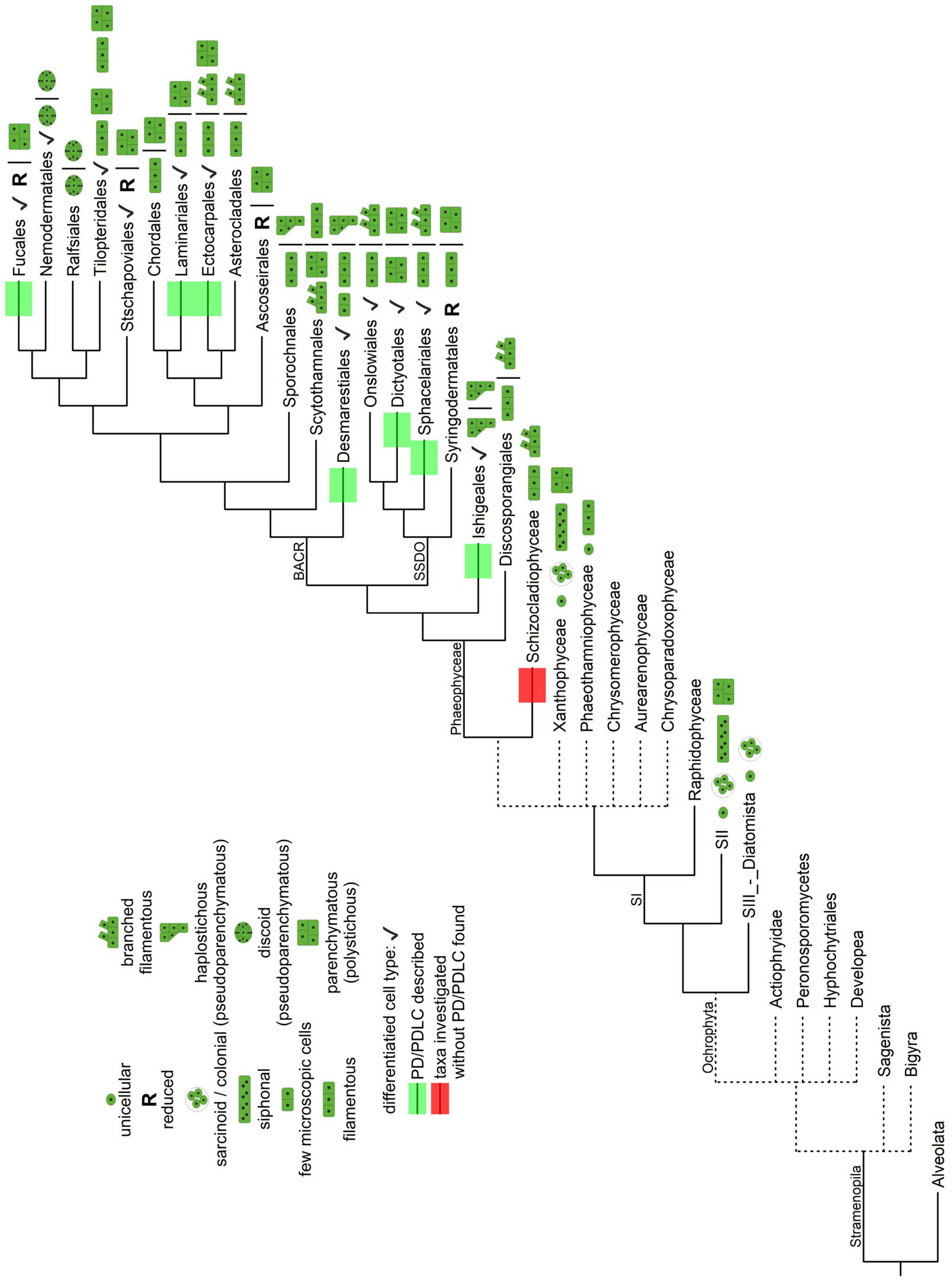
Fucales ✓ R
Nemodermatales ✓
Ralfsiales
Tilopteridales ✓
Stschapoviales ✓ R
Chordales
Laminariales ✓
Ectocarpales ✓
Asterocladales
Ascoseirales R
Sporochnales
Scytothamnales
Desmarestiales ✓
Onslowiales ✓
Dictyotales ✓
Sphacelariales ✓
Syringodermatales R
Ishigeales ✓
Discosporangiales
BACR
SSDO
Phaeophyceae
Schizocladiophyceae
Xanthophyceae
Phaeothamniophyceae
Chrysomerophyceae
Aurearenophyceae
Chrysoparadoxophyceae
Raphidophyceae
SI
SII
SIII_-_Diatomista
Ochrophyta
Actiophryidae
Peronosporomycetes
Hyphochytriales
Developea
Sagenista
Bigyra
Stramenopila
Alveolata
unicellular
R reduced
sarcinoid / colonial (pseudoparenchymatous)
siphonal
few microscopic cells
filamentous
branched filamentous
haplostichous
discoid (pseudoparenchymatous)
parenchymatous (polystichous)
differentiatied cell type: ✓
PD/PDLC described
taxa investigated without PD/PDLC found

**Figure S2:** Phylogeny of the Stramenopila (especially Phaeophyceae) with focus on the occurrence of PDLCs (color blocks), body plans (pictographs, indicated for gametophytes and sporophytes separated by `|´) and presence of cell differentiation (✓). The phylogeny is based on [77,106,110,130]. Body plan information and cell differentiation status is adopted from [130] with additional information from [5,77,110,151,288] The information on PD presence is based on Tables 1, 2, S1. Dotted branches indicate polytonies which are not sufficiently supported yet or groups whose exact relationship is not essential for the topic of this review. The branch lengths do not represent phylogenetic distances.

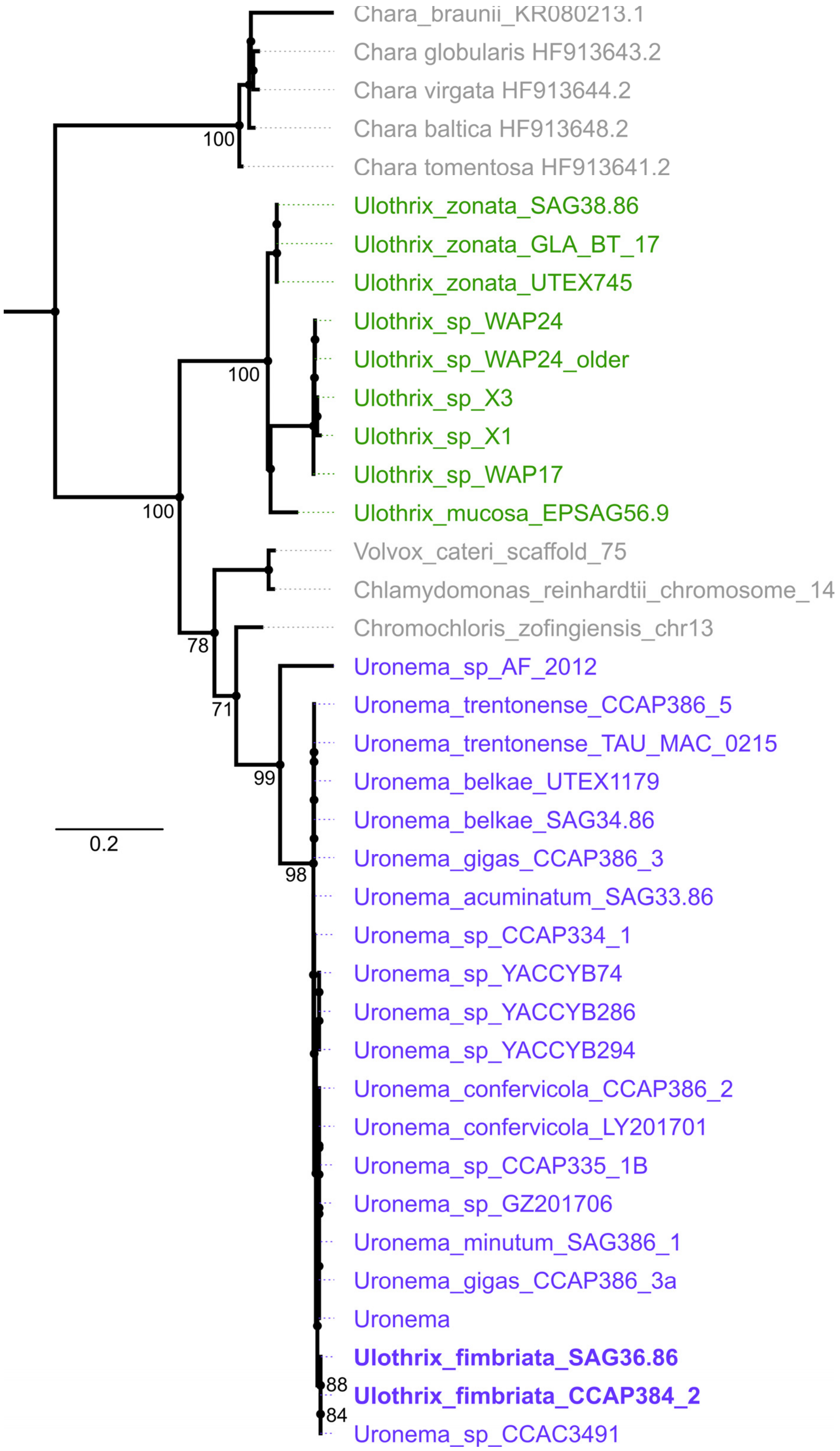


**Figure S3:** Midpoint rooted Maximum Likelihood phylogeny of 18S rRNA genes. Genomic sequences of 18S rRNA genes were retrieved from NCBI (*Ulothrix sp*. and *Uronema sp*.) and BLAST searches [223] using JGI Phytozome (*Volvox carteri*, *Chlamydomonas reinhardtii* and *Chromochloris zofingiensis*) [224] and NCBI (*Chara sp*.) [225]. Sequences were aligned using MAFFT (--localpair --maxiterate 10000 --reorder) [226]. Phylogeny (Maximum Likelihood, 100 bootstraps) was generated using iqtree (-T AUTO -m TEST -b 100 -con) [227]. (numbers show node support in %, *Ulothrix sp*. – green, *Uronema sp*. - blue, other – grey). The phylogeny was generated by Clemens Rössner JLU Giessen, Germany.

**Table S1:** List for the presence of PD/PDLCs and non-PD cell connection in Viridiplantae including current phylogenomic classification of species and studied strains (if traceable), mode of cell division (if mentioned), and inner structure of PD (if mentioned). If not mentioned otherwise in the comments column, algabase.org [110] was decisive for the determination of the current name and taxonomic classification. Direct quotes are in '…' and refer to paper within the same row if not mentioned otherwise. X: PD present; -: explicitly mentioned that no PD were found.
The corresponding references are listed in the second excel sheet. Publications were chosen according to the following criteria: explicit mention PD/PDLCs presence or absence, cited by others for PD presence/absence, extensive TEM studies including cellular interfaces (e.g. mostly cell division studies). Not all publications which do not show PD in the observed species are included if they do not explicitly state the lack PD/PDLCs or publications that could not be accessed. All mentions of PD should be covered (except for some repetitions by the same authors for the same species).

as extra file: Table_S1.xlsx
